# Ewing sarcoma breakpoint region 1 (EWSR1) protein binds to nonstructural protein 2 and non-coding regions of chikungunya virus genome to inhibit virus replication

**DOI:** 10.1128/jvi.00141-26

**Published:** 2026-07-21

**Authors:** Shivani Balyan, Anshula Sharma, Devendra Sharma, Deepti Jain, Sudhanshu Vrati

**Affiliations:** 1 Regional Centre for Biotechnology 214253, Faridabad, India; University of Michigan Medical School, Ann Arbor, Michigan, USA

**Keywords:** alphavirus, EMSA, BLI, NCR, RNA–protein binding

## Abstract

Chikungunya virus (CHIKV) is an alphavirus with a single-stranded RNA genome. Conserved sequence elements within the 5′ and 3′ non-coding regions (NCRs) of the CHIKV genome interact with host proteins to regulate viral RNA replication. Electrophoretic mobility shift assays (EMSAs) and mass spectrometry identified several host RNA-binding proteins that associate with these conserved NCR elements, among which EWSR1 uniquely bound the 3′ ends of both the plus-sense genomic RNA and the minus-sense replication intermediate RNA. EMSA and biolayer interferometry (BLI) demonstrated that EWSR1 binds strongly to two RNA elements: a 48-nucleotide region in the plus-strand 3′-NCR (3NCR48) and a 50-nucleotide region at the 3′-end of the minus-strand replication intermediate (5NCR-RC50), which is complementary to the genome’s 5′-NCR and its adjacent sequence. RNA immunoprecipitation from CHIKV-infected cells confirmed that EWSR1 binds CHIKV RNA *in vivo*. Functional analyses revealed that CHIKV infection downregulates EWSR1 expression and causes its accumulation in the cytoplasm. Knockdown or knockout (KO) of EWSR1 expression increased CHIKV genomic RNA levels and viral titers, whereas its overexpression suppressed both. EWSR1 reduced the synthesis of both plus- and minus-sense CHIKV RNAs in infected cells. Additionally, EWSR1 was found to bind to CHIKV nsP2 and inhibit its helicase activity, which is critical for viral genome replication. The antiviral activity of EWSR1 extended to Ross River virus but not to Sindbis virus, consistent with conservation of the 48-nucleotide sequence in their respective 3′-NCRs. Together, these findings identify EWSR1 as a novel host restriction factor that limits CHIKV replication through specific interactions with viral RNA and the nsP2 protein.

**IMPORTANCE:**

Chikungunya virus (CHIKV) poses a significant medical challenge, necessitating the development of novel antiviral strategies. This study demonstrates that the host protein EWSR1 plays a key role in controlling CHIKV replication. By binding to specific regions of viral RNA and interacting with nsP2, a critical viral enzyme, EWSR1 inhibits viral RNA synthesis and reduces viral titers. In the absence of EWSR1, CHIKV replicates more efficiently, underscoring its antiviral function. These findings establish EWSR1 as an important host restriction factor that limits CHIKV replication. Understanding this interaction enhances our knowledge of host–virus dynamics and may guide the design of new antiviral approaches against CHIKV and related alphaviruses.

## INTRODUCTION

Chikungunya virus (CHIKV) is a single-stranded plus-sense RNA genome containing an enveloped virus. It belongs to the Alphavirus genus of the *Togaviridae* family and is transmitted by the *Aedes* mosquitoes, primarily *Aedes aegypti* and *Aedes albopictus*. The virus causes chikungunya fever, characterized by a maculopapular rash, myalgia, arthralgia, nausea, and chills (1). CHIKV has caused periodic epidemics in Africa and Asia from the 1960s to the 2000s, a large-scale epidemic in the Indian Ocean region in 2005–2006, and a major outbreak in India in 2006 with 1.25 million cases. The virus has continued to cause frequent outbreaks across Asia, Africa, and the Americas, spreading to over 110 countries worldwide. In 2025, around 486,000 CHIKV cases and over 200 deaths were reported (2). Moreover, the virus causes severe, long-lasting indisposition in a large population each year. While a couple of CHIKV vaccines, IXCHIQ and VIMKUNYA, have recently been licensed (3–5), no antivirals are currently available. Understanding the virus replication and identification of critical events, such as the viral RNA interaction with the host protein/s, will greatly help in designing novel antivirals.

CHIKV has a ~11.8 kb genome having a 5′-methylguanylated cap and a 3′-polyA tail. The genome encodes four nonstructural and five structural proteins. The nonstructural proteins (nsP1, nsP2, nsP3, and nsP4) are synthesized from two-thirds of the genome toward its 5′-end. They are components of the replication complex required for genomic RNA (1) replication. The structural proteins (C, E3, E2, 6K, and E1) are synthesized from the sub-genomic RNA, comprising one-third of the genome toward the 3′-end (1). The CHIKV virion consists of an icosahedral nucleocapsid composed of the capsid protein and genomic RNA surrounded by a lipid envelope containing the E1 and E2 glycoproteins (6). While E1 induces pH-dependent fusion with the endosomal membrane, the E2 protein binds to cell surface attachment receptors (7).

The open reading frames in the CHIKV genome are flanked by 60- and 529-nucleotide non-coding regions (NCRs) at the 5′- and 3′-ends of the genome, respectively. The alphavirus NCRs contain conserved sequence elements (CSEs) that play an important role in the viral life cycle and the synthesis of the minus- and plus-sense viral RNAs (8). These NCRs interact with viral and host cell proteins to facilitate viral replication. A highly conserved AU dinucleotide at the very beginning of the 5′-NCR is essential for genome replication, likely serving as a recognition site for the viral RNA-dependent RNA polymerase (RdRp) during initiation of the plus-sense RNA synthesis (9–11). The 5′-NCR contains a stem-loop structure with a role in minus-strand RNA synthesis replication (9–11). It also contains core promoter elements necessary for initiation of both minus- and plus-sense RNA synthesis, along with other cis-acting RNA elements and 3′-UTR (11, 12). These elements are critical for the recruitment of the viral RdRp and other replication factors, including the viral and host proteins. Near the start of the nsP1 coding region (just downstream of the 5′-NCR), a 51-nucleotide CSE is present, which is important for virus replication, especially in mosquito cells (11).

The 3′-NCR also contains CSEs necessary for efficient viral RNA replication (11–13). Immediately upstream of the poly(A) tail, a short, highly conserved sequence (19–24 nucleotides) is present in all alphaviruses. This element is essential for viral replication and acts as a core promoter for minus-sense RNA synthesis (14). The 3′-NCR contains multiple short repeat sequence elements (RSEs), which are often arranged in tandem and vary in copy number among different alphavirus species. These RSEs bind host proteins and are important for efficient virus replication (11, 12, 14). A U-rich sequence of ~60 nucleotides is often found upstream of the conserved 19-nucleotide region. This domain interacts with host proteins to stabilize the viral genome (14). Recent studies have identified a highly conserved Y-shaped stem-loop structure within the CHIKV 3′-NCR that plays roles in replication efficiency and possibly in evading host defenses (15).

Several host proteins have been identified that bind the alphavirus NCRs. The 5′-NCR interacts with host translation initiation factors eIF4E and eIF4F, which are critical for initiating protein synthesis (11). Heterogeneous nuclear ribonucleoprotein A1 (hnRNP A1) binds to the 5′-NCR of Sindbis virus (SINV) RNA and enhances the synthesis of minus-sense RNA (16). The cellular HuR protein that binds to the U-rich elements of the 3′-NCR of SINV RNA and upstream of the 3′-CSE in the 3′-NCR of Ross River virus (RRV) is important for viral genome stability and its efficient replication (11, 17, 18). The La autoantigen also interacts with the 3′-NCR, likely contributing to RNA stability and possibly influencing translation or replication (11, 19).

Collectively, these studies illustrate the pivotal role of CSEs and their interaction with host proteins in alphavirus replication. In the present work, several host proteins that specifically recognize CSEs in the CHIKV NCRs have been identified, with a focus on determining the functional impact of EWSR1 in CHIKV replication.

## MATERIALS AND METHODS

### Cells and viruses

Human embryonic kidney cells (HEK293T) and embryonal rhabdomyosarcoma (ERMS) cells were grown in Dulbecco’s modified Eagle’s medium (DMEM) (HiMedia, AL007A) with 10% fetal bovine serum (FBS) (Gibco, 10270106). African green monkey kidney cells (Vero) and baby hamster kidney cells (BHK-21) were cultured in minimum essential medium (MEM) (HiMedia, AL0475) with 10% FBS. HAP1 cells (a near-haploid human cell line derived from the KBM-7 chronic myelogenous leukemia cell line) were grown in Iscove’s modified Dulbecco’s medium (IMDM) (HiMedia, AL070S) with 10% FBS. All cells were grown at 37°C in a humidified atmosphere with 5% CO_2_. All cultures were supplemented with 1× Penicillin-Streptomycin-Glutamine solution (HiMedia, A001A). The IND-06 Guj strain of CHIKV (GenBank accession number JF270482.1), the EG AR 1019 strain of SINV, and the T48 strain of RRV (GenBank accession number GQ433359.1) were used in this study. The IND-06 Guj strain of CHIKV was isolated in the lab from a CHIKV patient of the 2006 Gujarat outbreak. SINV and RRV were obtained from the World Reference Center for Emerging Viruses and Arboviruses, University of Texas Medical Branch, USA. The viruses were grown in BHK-21 cells, and their titers were determined by plaque assay.

### Plaque assay

Vero cells were seeded in 6-well tissue culture plates overnight to achieve monolayers with approximately 80% confluency. The cells were infected with serial dilutions of virus-containing culture supernatants prepared in incomplete MEM. After 1 h of incubation, the cells were washed with 1× PBS and incubated at 37°C with an overlay of 2% low-melting agarose (Sigma-Aldrich, A4018) and an equal volume of 2× MEM supplemented with 10% FBS and 1× penicillin-streptomycin-glutamine solution (HiMedia, A001A) for 36 h. The cell monolayers were then fixed with 4% formaldehyde and stained with 0.2% crystal violet to visualize the plaques.

### Electrophoretic mobility shift assay

Electrophoretic mobility shift assay (EMSA) was performed as described previously with slight modifications (20). HEK293T, Vero, and ERMS cells were harvested and lysed using a lysis buffer containing 25 mM Tris, pH 7.4, 150 mM NaCl, 1 mM EDTA, 1% NP-40, and 5% glycerol. The protease inhibitor cocktail (Sigma-Aldrich, P8340) was added immediately before cell lysis. The lysates were incubated on ice for 1 h with periodic vortexing and spun down, and the supernatant was collected for protein estimation by the Bradford Assay kit (Thermo Scientific, 23200). Cell lysate containing 10 μg protein was incubated with 10 μg poly(I)-poly(C) (polyIC) (dsRNA mimic) (Merck, P1530), 20 ng biotin-labeled synthetic RNA, and 1 U RNasin (Promega, N2611) in the binding buffer (14 mM HEPES, pH 7.5, 6 mM Tris-Cl, pH 7.5, 1 mM EDTA, 1 mM DTT, and 60 mM KCl) in a final volume of 25 μL for 30 min at room temperature with shaking. PolyIC was used as a nonspecific competitor RNA to assess the specificity of RNA–protein interactions (20–22). The RNA–protein mixture was then crosslinked using a UV torch (4 watts) for 30 min. The RNA–protein complexes were electrophoresed on a nondenaturing 12% polyacrylamide gel (acrylamide/bisacrylamide ratio 29:1) at 4°C in 0.5× Tris-borate-EDTA buffer. The gel was transferred to a Bio-Dyne nylon membrane (Thermo, 77016) at 200 mA, 4°C for 2 h. The membrane was then developed using a chemiluminescent nucleic acid detection module kit as per the manufacturer’s protocol (Thermo, 89880), in which the blot was blocked and subjected to streptavidin-HRP conjugate, and then HRP was detected using luminol enhancer and visualized on X-ray film. The nucleotide sequences of the synthetic RNAs used in the study are provided in Table S1.

### RNA pull-down and mass spectrometry

Cell lysate containing 2 mg protein was incubated in binding buffer (14 mM HEPES, pH 7.5, 6 mM Tris-Cl, pH 7.5, 1 mM EDTA, 1 mM DTT, and 60 mM KCl) with 2 mg polyIC, 4 μg synthetic RNA, and 1 U RNasin in a final volume of 2 mL for 45 min at room temperature with shaking. After this, the RNA–protein complexes were crosslinked with a UV torch for 30 min. Dynabeads MyOne streptavidin C1 (Invitrogen, 65001) beads were washed thrice with binding buffer. The RNA–protein mixture was added to the beads and incubated for 30 min at room temperature with shaking. Beads were then washed with binding buffer, and bound proteins were eluted using 95% formamide and 10 mM EDTA at 65°C for 5 min. The eluted proteins were then buffer exchanged with 50 mM ammonium bicarbonate, alkylated using iodoacetamide, digested with 1 μg trypsin (Promega, V5280) at 37°C for 18 h, and analyzed by ESI-MS using an LC/MS (SCIEX Triple TOF 5600+). Data were collected from three independent experiments, each with two technical replicates (Fig. S1). Data-dependent acquisition (DDA) was used for peptide sequencing and protein identification qualitatively. All raw files were searched using ProteinPilot software (version 5.0.2) against the Swiss-Prot database R 6.12.2017. An unused score threshold of 1.3 was applied, giving >95% confidence, and only proteins identified with a minimum of two unique peptides in the experimental samples (beads + RNA + protein or beads + RNA + protein + polyIC) were retained for further analyses. Proteins detected with a single peptide in the control samples (beads + protein) and proteins detected in the same way with Random48 RNA were excluded (Fig. S1). Proteins detected in at least four out of six total analyses (three biological × two technical replicates) were finally cataloged as the potential binders. The mass spectrometry data have been submitted to the Indian Biological Data Centre (IBDC) on the Indian Proteome Databank (IPD) portal (accession ID: IPD 9067).

### siRNA transfection and virus infection of the cells

ON-TARGETplus SMARTpool small interfering RNAs (siRNAs) targeting EWSR1 and a nontargeting control were obtained from Dharmacon. HEK293T cells were transfected with 30 nM siRNA using DharmaFECT1 (Horizon Discovery, T-2005-01) following the manufacturer’s instructions. At 48 h post-transfection, cells were infected with CHIKV, RRV, or SINV and harvested at different hours post-infection (hpi). Cells and supernatant were subsequently processed for western blotting, qRT-PCR, and plaque assays, respectively.

### Generation of the knockout HAP1 cells

HAP1 cells were transfected with the CRISPR/Cas9 EWSR1 knockout plasmid synthesized by GeneScript. The knockout plasmid was based on the GenScript plasmid eSpCas9-2A-Puro (PX459) V2.0. The sgRNA sequence (sgEWSR1: 3′-TACACCGCCCAGCCCACTCA-5′) was selected from the GeCKO v2 Human CRISPR Knockout Pooled Library and reviewed by the CHOPCHOP web tool. The sgRNA/Cas9 plasmid was transfected using Lipofectamine 3000 (Thermo, L3000001). The transfected cells were selected using puromycin solution (InvivoGen; ant-pr-1) at a 0.8 µg/mL concentration. The absence of EWSR1 expression in the KO cells was confirmed by western blotting.

### EWSR1 ectopic expression studies

The full-length EWSR1 cDNA was amplified using total cellular RNA and the following synthetic oligonucleotide primers: forward primer- ATGCGTCGACATGGCGT CCACGGAT (Sal I restriction site underlined) and reverse primer- ATATGCGGCCGCCTAGTAGGGCCGATCT (Not I restriction site underlined). The cDNA was cloned into the pCMV Script vector (Novopro, V011243) under the CMV promoter. HEK293T cells were transfected with the plasmid using Lipofectamine 3000 according to the manufacturer’s protocol. At 24 h post-transfection, cells were infected with CHIKV, RRV, or SINV and harvested at 12 hpi. Cells and supernatant were then processed for western blotting, qRT-PCR, and plaque assay, respectively.

### Nuclear and cytoplasmic fractionation of cell lysate

HEK293T cells were infected with CHIKV and harvested at 12 hpi. Nuclear and cytoplasmic fractions were obtained using the NE-PER nuclear and cytoplasmic extraction reagents (Thermo Fisher, 78833) as per the manufacturer’s protocol. GAPDH and Lamin A/C were used to establish the purity of cytoplasmic and nuclear fractions, respectively.

### RNA isolation and quantitative real-time PCR

Total RNA was isolated from the cells using the RNAiso Plus reagent (Takara, 9109) and purified using the phenol-chloroform method. First-strand cDNA was synthesized using 100 ng total RNA with the ImProm II Reverse Transcriptase kit (Promega, A3802). Quantitative real-time PCR (qRT-PCR) was set up using the TB Green Premix Ex Taq (Takara; RR420A). The reactions were performed using QuantStudio 6 (Applied Biosystems). The following primers were used: CHIKV forward- AAGCTYCGCGTCCTTTACCAAG, CHIKV reverse- CCAAATTGTCCYGGTCTTCCTG; SINV forward- AAAGGATACTTTCTCCTC GC, SINV reverse- TGGGCAACAGGGACCATGCA; RRV forward- GCGACGGTGGAT GTCAAGGAG, RRV reverse- AGCCAGCCCACCTAACCCACTG; GAPDH forward- GGTGAAGGTCGGAGTCAACG, GAPDH reverse- AGGGATCTCGCTCCTGGAAG; EWSR1 forward- GGGTATGGCACTGGTGCTTAT, and EWSR1 reverse- CAGACTGAGCTG CATAGGAGG. The GAPDH RNA levels were used as the internal control for normalization.

### RNA immunoprecipitation

HEK293T cells were infected with CHIKV and harvested at 12 hpi. RNA immunoprecipitation (RIP) was performed using the MAGNA RNA Binding Protein RIP kit (Sigma-Aldrich, 17-700) according to the manufacturer’s instructions. Cells were lysed in RIP lysis buffer, and lysate containing an equal amount of protein was incubated with either EWSR1 antibody or an isotype-matched IgG antibody bound to protein A/G magnetic beads. After washing, RNA–protein complexes were treated with proteinase K to digest the protein, and RNA was purified using the phenol-chloroform method. qRT-PCR to determine CHIKV RNA levels was performed as described earlier. Using previously published protocols (23–25), Ct values were normalized to GAPDH, and relative fold changes were calculated with respect to the IgG control. The strand-specific quantitation of CHIKV plus-sense RNA was done as described below.

### Recombinant EWSR1 protein expression and purification

Full-length EWSR1 cDNA was amplified using total cellular RNA and the following synthetic oligonucleotide primers: forward primer- ATGCCGGAATTCGCGTCCACGGATTAC with EcoRI site (underlined) and reverse primer- ATATGCGGCCGCCTAGTAGGGCCGATCTC with NotI site (underlined). The cDNA was cloned under the T7 promoter into the pET28a (Novagen, 69864) vector. *Escherichia coli* BL21-DE3 cells transformed with the pET28a-6xHis-EWSR1 plasmid were grown in Luria-Broth at 37°C until absorbance A_600_ reached 0.6–0.8. The recombinant protein expression was then induced by the addition of 0.5 mM isopropyl-β-d–thiogalactoside (IPTG), followed by incubating the cells under shaking at 22°C for 14 h. For the purification of 6xHis-tagged EWSR1 protein, cell pellets were centrifuged at 4,000 × *g* at 4°C for 20 min and resuspended in lysis buffer containing 50 mM Tris–HCl, pH 8.0, 500 mM NaCl, 5 mM EDTA, 1 mM PMSF, and then lysed by sonication (Sonics and Materials, 13 mm probe, 55% amplitude, 10 cycles with pulses of 10 s on and 50 s off). The cell lysates were centrifuged at 16,000 × *g* for 20 min at 4°C. The pellet was resuspended in wash buffer A (50 mM Tris-Cl, pH 8, 500 mM NaCl, 1% Triton X-100, 5 mM EDTA, and 1 mM PMSF) and then sonicated again. The cell lysates were centrifuged at 16,000 × *g* for 20 min at 4°C, and the pellet was homogenized in wash buffer B (50 mM Tris-Cl, pH 8, 500 mM NaCl, and 1 mM PMSF). This was followed by centrifugation at 16,000 × *g* for 20 min at 4°C. The pellet was then solubilized in 50 mM Tris-Cl, pH 12, 500 mM NaCl, 5 mM EDTA, 1 mM PMSF, 2 M urea, and incubated at room temperature for 1 h with periodic vortexing, and then centrifuged at 16,000 × *g* for 30 min. Supernatant containing EWSR1 was then allowed to refold in 50 mM Tris-Cl, pH 8, 10% sucrose, 1 mM EDTA, and 1 mM PMSF for 14 h at 4°C. The refolded sample was then centrifuged at 16,000 × *g* for 30 min. Supernatant was then loaded onto a pre-equilibrated Ni-NTA column and washed with the washing buffer containing 20 mM Tris–HCl, pH 8.0, 500 mM NaCl, and 20 mM imidazole, followed by elution with the elution buffer containing 20 mM Tris-HCl, pH 8.0, 500 mM NaCl, and 200 mM imidazole (26). The eluted protein was quantified using the Bradford Assay Kit (Thermo Scientific, 23200). The protein identity and purity were established by electrophoresis on a 12% SDS-PAGE followed by western blotting, as well as by peptide mass fingerprinting.

### Expression and purification of CHIKV nsP2 protein

The full-length CHIKV nsP2 protein, as well as its helicase and protease domains, were expressed and purified as described previously (27). Briefly, a modified pET14b expression plasmid encoding the gene of interest and an N-terminal His_6_-SUMO tag was used to transform *Escherichia coli* Rosetta (DE3) cells. Cultures were grown in LB to an OD_600_ of 0.6–0.8 and induced with 0.5 mM IPTG at 18°C for 16 h. Cells were harvested and lysed in a buffer containing 20 mM HEPES, pH 7.5, 500 mM NaCl, 5% glycerol, and 2 mM β-mercaptoethanol. The lysate was clarified and applied to a Ni-NTA column (Cytiva), followed by elution using an imidazole gradient. His_6_-SUMO tag was removed using PreScission protease, and the sample was dialyzed overnight at 4°C. The protein was concentrated and subjected to size-exclusion chromatography using a Superdex 200 16/600 (Cytiva) column in 20 mM HEPES, pH 7.5, 300 mM NaCl, 5% glycerol, and 0.5 mM DTT. The purity was confirmed by SDS-PAGE.

### Western blotting

The cells were harvested and lysed using a lysis buffer containing 25 mM Tris, pH 7.4, 150 mM NaCl, 1 mM EDTA, 1% NP-40, and 5% glycerol. The protease inhibitor cocktail (Sigma-Aldrich, P8340) was added immediately before cell lysis. The cell lysates were incubated on ice for 1 h with periodic vortexing and spun down, and the supernatant was collected for protein estimation by the Bradford Assay kit (Thermo Scientific, 23200). The 5× SDS-PAGE loading dye was then added to the protein lysates and heated at 95°C for 10 min. Equal amounts of protein lysates were then resolved on a 12% SDS-PAGE and transferred onto a polyvinylidene difluoride (PVDF) membrane. The membrane was then blocked with 5% BSA for 1 h at room temperature, followed by overnight incubation with the primary antibody at 4°C. The membrane was then washed with PBS with 0.1% Tween-20 and incubated with a horseradish peroxidase (HRP)-conjugated secondary antibody for 1 h. The protein bands were visualized using the SuperSignal West Pico PLUS Chemiluminescent substrate (Thermo Scientific; 34577). ImageJ software was used to measure the EWSR1 protein band intensity and normalized to that of GAPDH or Lamin, as mentioned in the figure legends.

The following antibodies were used in the study: rabbit anti-GAPDH (Genetex, GTX100118), rabbit anti-lamin (Thermo Fisher Scientific, MA5-35284), rabbit anti-capsid (Genetex, GTX135183), rabbit anti-EWSR1 (Cell Signaling Technology, 11910), rabbit anti-CHIKV nsP2 (Genetex, GTX636897), and HRP-conjugated anti-rabbit secondary antibody (Invitrogen, 65-6120).

### Biolayer interferometry

The binding of EWSR1 to RNAs was studied using the ForteBio Octet Red instrument. The ligand, biotinylated-RNA (50 ng) in nuclease-free water, was loaded on the surface of a streptavidin-conjugated biosensor for 600 s. The binding assays were performed in black 96-well plates at 25°C, under agitation (1,000 rpm), in 200 µL binding buffer (14 mM HEPES, pH 7.5, 6 mM Tris-Cl, pH 7.5, 1 mM EDTA, and 60 mM KCl). Initially, a 120-s biosensor baseline step was performed in buffer only. Following this, association of the biosensor-bound RNA ligand with the EWSR1 protein (0–800 nM) in the binding buffer was recorded for 180 s. Finally, dissociation was allowed for 180 s. For the CHIKV nsP2/EWSR1 interaction, the histidine-tagged EWSR1 (10 µg) was loaded on the surface of a Ni-NTA biosensor for 300 s. The sensor loaded with EWSR1 was allowed to associate with CHIKV nsP2 (100–1,000 nM) for 400 s in 50 mM Tris buffer (pH 7.4). The dissociation was then followed for 800 s. Correction of any systematic baseline drift was done by subtracting the shift recorded for the sensor loaded with the ligand in the absence of analyte. The BLI sensorgram was plotted showing the biosensor’s response against time for different analyte concentrations. The data were fitted to a 2:1 binding model to determine the equilibrium dissociation constant (*K*_d_). Only data with an *R*^2^ value of >0.95 were included in the analysis.

### Strand-specific quantitation of CHIKV RNA

The plus- and minus-sense RNAs of CHIKV were quantified using a strand-specific qRT-PCR assay as described previously (27). Briefly, total RNA isolated from CHIKV-infected cells was employed for cDNA synthesis by reverse transcription. Tagged (non-viral sequence) primers PtagCHIKp and NtagCHIKn were used for cDNA synthesis from the plus- and minus-sense CHIKV RNA, respectively. Real-time qPCR was performed using a combination of primers that bind to the non-viral tag sequence and the viral strand. The strand-specific RNA copy numbers were determined using the plus- and minus-sense RNA-specific standard curves.

### Co-immunoprecipitation

HEK293T cells were infected with CHIKV for 12 h. The cells were washed with 1× PBS and lysed using RIPA lysis buffer. The CHIKV proteins were co-immunoprecipitated from 1 mg cellular protein lysate and 5 µg rabbit anti-EWSR1 (Cell Signaling Technology, 119100) antibody or rabbit IgG as an isotype control using the co-immunoprecipitation kit (Thermo Scientific; 26149). The immunoprecipitated proteins were Western blotted using the CHIKV anti-nsP2 antibody (Genetex; 636897) or rabbit anti-EWSR1 antibody (Cell Signaling Technology, 119100).

### Helicase assay

To investigate the effect of EWSR1 on the RNA unwinding activity of CHIKV nsP2, a helicase assay was performed following the protocol described previously (28, 29). The assay utilized an Alexa Fluor 488-labeled 28-mer ssRNA oligonucleotide (Alexa488-ssRNA, 5′-AAAAAAAAAAAACCAGGCGACAUCAGCG-3′) and an unlabeled 16-mer ssRNA oligonucleotide (5′-CGCUGAUGUCGCCUGG-3′) to generate a dsRNA helicase substrate with a 12-base 5′ overhang. To prepare the dsRNA substrate, the labeled and unlabeled RNA oligonucleotides were mixed at a 1:1.1 molar ratio in a buffer containing 10 mM HEPES (pH 7.2) and 20 mM KCl. To facilitate annealing, the RNA mixture was heated to 95°C for 1 min in a thermal cycler, followed by gradual cooling at a rate of 1°C per min until reaching 22°C. The optimized reaction mixture for the unwinding assay consisted of the strand-displacement assay buffer (40 mM HEPES, pH 7.5, 2 mM dithiothreitol [DTT], and 12 mM NaCl), 1 μM purified CHIKV nsP2 protein, 50 nM dsRNA substrate, 800 nM unlabeled RNA trap (5′-CCAGGCGACAUCAGCG-3′), 20 U RNaseOUT inhibitor (Thermo Fisher Scientific, 10777019), and varying concentrations of EWSR1 protein. The reaction mixture was incubated at 26°C for 20 min in a thermal cycler to facilitate the formation of the protein–RNA complex. Subsequently, a 3.5 mM ATP-magnesium acetate mixture was added, followed by incubation at 37°C for 120 min. The reactions were then terminated by adding a stop solution containing 100 mM Tris-HCl, pH 7.5, 0.1% bromophenol blue, 1% SDS, 50 mM EDTA, and 50% glycerol. The separation of ssRNA and dsRNA was carried out through electrophoresis on a 15% nondenaturing polyacrylamide gel at 4°C. The RNA bands were visualized using a Typhoon imager (Cytiva), and their intensities were quantified using the ImageJ software.

### CHIKV nsP2 protease assay

The proteolytic activity of CHIKV nsP2 protease was determined using a FRET-based approach (27, 30, 31). The assay was performed *in vitro* using the purified CHIKV nsP2 protein and the FRET-based octapeptide substrate {DABCYL}-RAGG↓YIFSS-{Glu(EDANS)}-NH2 (Biolink) representing the nsP3/4 site (32). For the assay, 1 μM CHIKV nsP2 was incubated with different concentrations of EWSR1 in the assay buffer (20 mM Bis-Tris-Propane, pH 8) for 30 min at 25°C. Following the incubation, the nsP2-EWSR1 mix was added to a Nunc 96-well black plate (Thermo Fisher Scientific, 137101) with 25 μM peptide substrate in the assay buffer, and the fluorescence was measured at 30-s intervals using a multimode plate reader, SpectraMax i3x, at an excitation wavelength of 340 nm and an emission wavelength of 490 nm. A substrate control reaction measured the auto-fluorescence generated by just the substrate. Withanone was used at 10 μM concentration as a negative control, and withaferin A at 10 μM concentration as a positive control for the protease activity inhibition assay (27). All enzyme reactions were performed in duplicate and in two independent experiments. The average fluorescence of each sample was calculated and plotted in the graph.

### Statistical analysis

The statistical significance of differences in the means of multiple data values was determined by unpaired Student’s t-test and is indicated in the figures as *, *P* < 0.05; **, *P* < 0.01; ***, *P* < 0.001; ****, *P* < 0.0001; ns = not significant.

## RESULTS

### CHIKV genome has alphavirus-conserved genome nucleotide sequences

**Fig 1:**
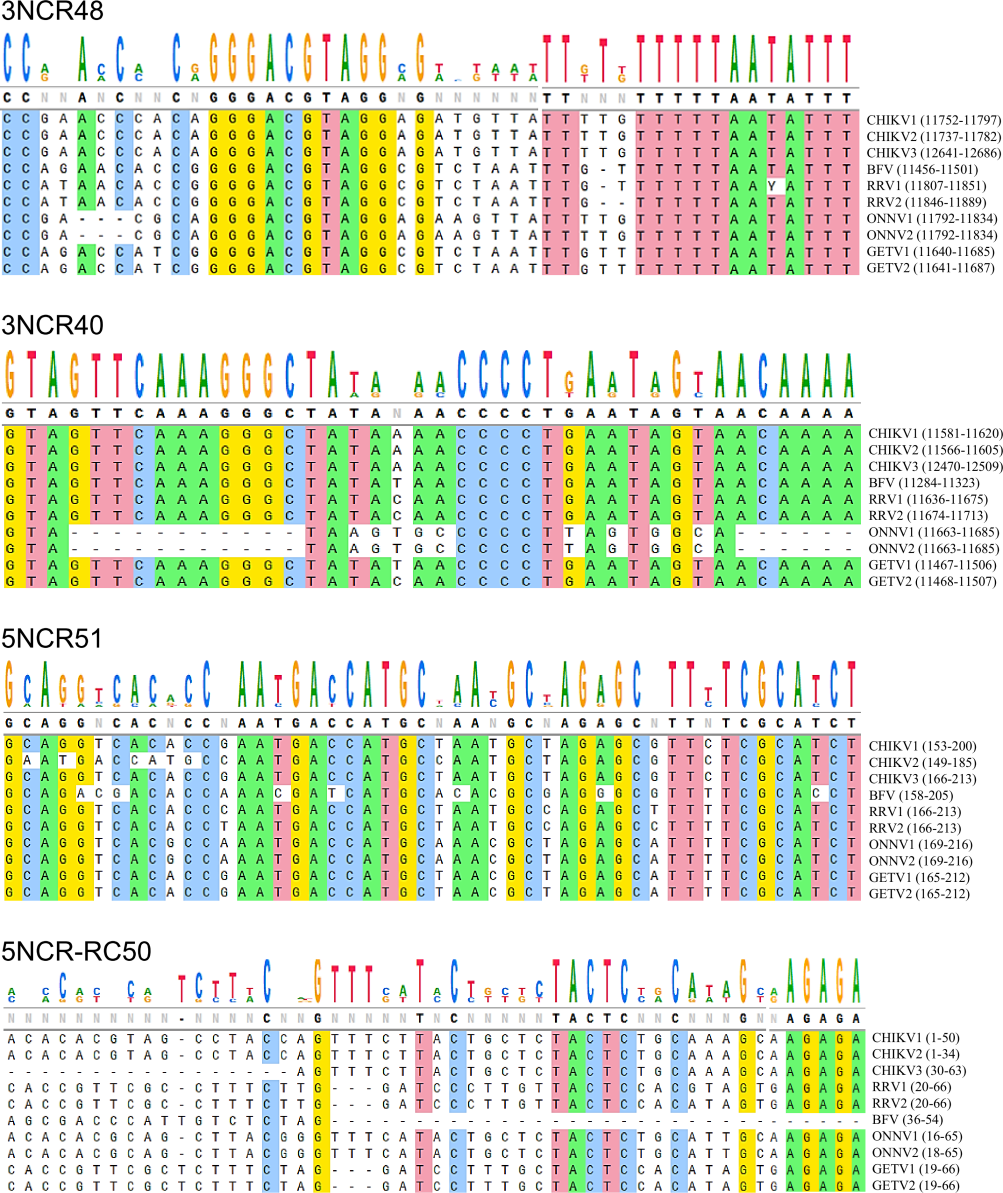
Conserved sequences in the alphavirus genomes. The 3′- and 5′-NCR sequences in CHIKV-related arthritogenic alphaviruses, such as RRV, ONNV, GETV, and BFV, were aligned using Clustal software. The figure shows the alignment to demonstrate sequence conservation in the 3′-NCR around the CHIKV sequence represented in 3NCR48 and 3NCR40 sequences, and in the 5′-NCR around CHIKV sequences represented in 5NCR51 and 5NCR-RC50. The GenBank accession numbers of the various sequences used for alignment are as follows: CHIKV1, JF274082.1; CHIKV2, MH349097.1; CHIKV3, EU224269.1; BFV, MK697274.1; RRV1, GQ433359.1; RRV2, NC_075016.1; ONNV1, M20303.1; ONNV2, NC_001512.1; GETV1, LC814433.1; GETV2, MT086508.1. The location of the aligned sequences on the respective genomes as nucleotide numbers is shown in brackets.

Three previously reported alphavirus-conserved genomic RNA sequences (8, 11–13) were examined for their conservation in the CHIKV RNA genome. The 40-nucleotide 3NCR40 sequence (nucleotide 11,581–11,620) is part of the conserved elements and RSEs found upstream in the 3′-NCR. The 48-nucleotide 3NCR48 sequence represents the extreme 3′-end sequence of the CHIKV 3′-NCR (nucleotides 11,751–11,798). The 51-nucleotide 5NCR51 located from nucleotides 153 to 203 in the CHIKV nsP1-coding region is downstream of the 44-nucleotide putative minus-sense RNA promoter and is critical for efficient virus replication (15, 33, 34). The 50-nucleotide 5NCR-RC50 has sequence complementary to the 5′-NCR sequence and may contain elements required for plus-sense genomic RNA synthesis (9, 11, 35). Alignment of these CHIKV sequences showed 54%–98% sequence similarity across CHIKV-related arthritogenic alphaviruses, such as RRV, O'nyong-nyong virus (ONNV), Getah virus (GETV), and Barmah Forest virus (BFV) (Fig. 1; Table S2). The 3NCR48 RNA sequence had the highest sequence similarity (79%–90%) among the different alphaviruses. For the 3NCR40 sequence, the sequence similarity was 57%–98%, whereas it was 75%–94% for 5NCR51, and 57%–86% for 5NCR-RC50 across different alphaviruses. A high level of sequence conservation (98%–100% similarity) was seen among different CHIKV strains (Table S2).

### Host proteins bind to conserved CHIKV RNA sequences

**Fig 2:**
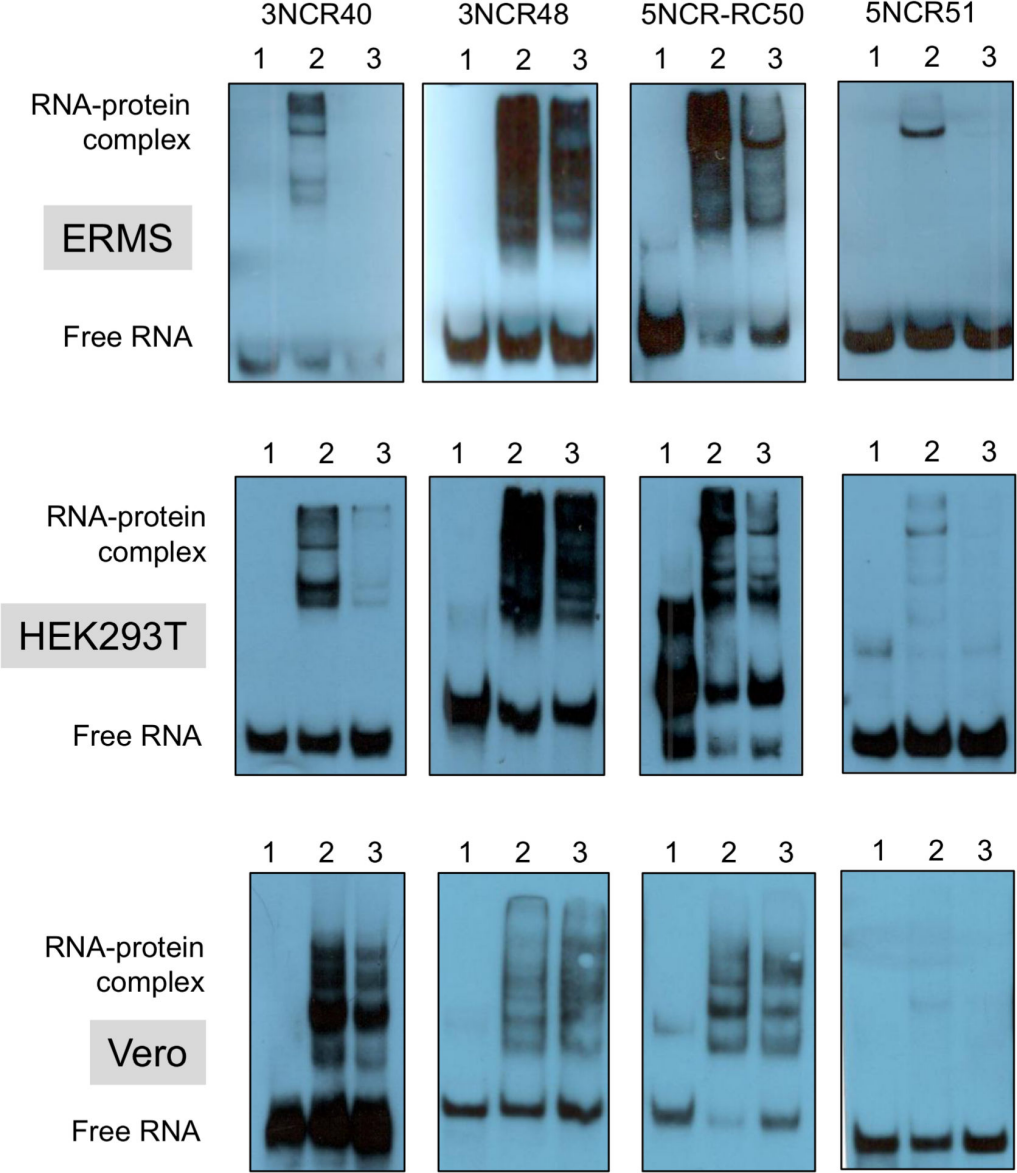
Host proteins bind to conserved CHIKV RNA sequences. EMSA was used to study the binding of ERMS, HEK293T, and Vero cell proteins to 3NCR40, 3NCR48, 5NCR-RC50, and 5NCR51. Biotin-labeled synthetic RNA (20 ng) was incubated in the binding buffer with 10 μg cellular protein lysate in the presence or absence of polyIC. The RNA–protein complexes were electrophoresed on a nondenaturing 12% polyacrylamide gel and transferred to a membrane. The RNA and the RNA–protein complexes were visualized using a chemiluminescent nucleic acid detection method. Lane 1, RNA; lane 2, RNA–protein complexes in the absence of polyIC; lane 3, RNA–protein complexes in the presence of 10 μg polyIC.

EMSA was used to study the binding of proteins from ERMS, HEK293T, and Vero cells to different CHIKV RNAs. While a number of host proteins from these cells showed binding to 3NCR40, 3NCR48, and 5NCR-RC50, very little host protein binding was seen with 5NCR51 (Fig. 2). Some of the RNA–protein bands diminished or disappeared in the presence of excess polyIC, indicating nonspecific or weak binding. PolyIC was used as a nonspecific RNA competitor. However, several RNA–protein bands were not affected even in the presence of excess polyIC, indicating a high-affinity, specific binding of the protein to the CHIKV RNAs. Importantly, the binding profile was different for protein lysates from different cells. The binding profile of the protein lysate from a given cell line was also different for different RNAs. These data suggest that different host cell proteins may bind to different CHIKV conserved RNA sequences, and different proteins from different cells may bind to a given RNA sequence.

### Identification of host proteins binding to CHIKV RNA sequences

Host proteins interacting with the biotin-labeled CHIKV RNAs were pulled down from host cell lysates using streptavidin beads, and mass spectrometry was used to identify the proteins. The specific proteins binding to the CHIKV RNAs were identified by eliminating those proteins that bind to the beads or a random RNA (Fig. S1). The host proteins identified at least four times from the six experimental replicates are listed in Table 1. It can be seen that a diverse set of proteins were binding to different CHIKV RNAs. As seen in the EMSA, different proteins from different cells were binding to a given RNA sequence, except for CPSF7, which bound 3NCR48 in Vero, ERMS, and HEK293T cell lysates, and QKI, which bound 3NCR40 in both ERMS and HEK293T cell lysates. Notably, only a smaller number of proteins were binding to 5NCR51 RNA. It should be noted that multiple technical factors, including sample preparation, detection limits, and sampling in mass spectrometry, can contribute to differences in protein identification. Nonetheless, diversity in identified proteins indicates the specificity of the RNA–protein binding.

**TABLE 1:** Host proteins binding to CHIKV RNAs^*a*^

CHIKV RNA	Vero cells	ERMS cells	HEK293T cells
3NCR40	ROA3 (4/6) QOR (4/6) DAZP1 (4/6)	QKI (6/6)	QKI (6/6) PSPC1 (6/6) HNRDL (6/6) ROA0 (6/6)
3NCR48	CPSF7 (6/6) ELAV1 (6/6) PUF60 (6/6) RBMS1 (6/6) ROA0 (6/6) SRSF9 (6/6) TIA1 (6/6)	CPSF6 (6/6) CPSF7 (6/6)	EWSR1 (6/6) CPSF6 (6/6) CPSF7 (6/6) HNRH3 (6/6)
5NCR51	IMDH2 (6/6)	SSBP1 (5/6)	CAPR1 (4/6)
5NCR-RC50	FUS (6/6)	HNRDL (6/6)	EWSR1 (6/6) G3BP1 (6/6)

^*a*^ The number of times the protein was identified out of the multiple experimental replicates is shown in parentheses.

The PANTHER analysis was used as a functional classification tool to annotate the molecular functions of the proteins identified in the RNA pull-down interactome (Table 2). This analysis revealed that most of the identified proteins were annotated with nucleic acid–binding functions, especially RNA binding. This enrichment of RNA-binding proteins (RBPs) validated the RNA pull-down approach used in the study.

**TABLE 2:** PANTHER analysis of CHIKV RNA-binding proteins

GO molecular function	Proteins
RNA binding	ROA3, QOR, DAZP1, QKI, PSPC1, HNRDL, ROA0, CPSF7, ELAV1, PUF60, RBMS1, SRSF9, TIA1, EWSR1, CPSF6, HNRH3, IMDH2, SSBP1, CAPR1, FUS, HNRDL, and G3BP1
mRNA binding	ROA0, CAPR1, TIA1, CPSF6, FUS, ROA3, G3BP1, RBMS1, ELAV1, QKI, QOR, DAZP1, and CPSF7
mRNA 3′-NCR binding	ROA0, TIA1, FUS, ROA3, RBMS1, ELAV1, QKI, QOR, and DAZP1
Nucleic acid binding	ROA0, CAPR1, HNRDL, TIA1, PSPC1, CPSF6, FUS, IMDH2, EWSR1, PUF60, ROA3, G3BP1, RBMS1, ELAV1, SSBP1, SRSF9, QKI, HNRH3, QOR, DAZP1, and CPSF7
Poly-purine tract binding	HNRDL, TIA1, RBMS1, and DAZP1
Single-stranded RNA binding	HNRDL, TIA1, RBMS1, and DAZP1
Poly(A) binding	HNRDL, TIA1, and RBMS1
mRNA 3′-NCR AU-rich region binding	ROA0, TIA1, and ELAV1
Molecular condensate scaffold activity	CAPR1, FUS, and G3BP1
Poly(G) binding	HNRDL and DAZP1

Interestingly, EWSR1, detected in all six experimental replicates, was interacting with both the plus- and minus-sense CHIKV RNAs, represented by 3NCR48 and 5NCR-RC50, respectively, in HEK293T cell lysate. EWSR1 is a 656-residue multifunctional RNA/DNA-binding protein with a role in regulating transcription, splicing, and mRNA transport (36). EWSR1 supports hepatitis C virus (HCV) replication by binding to the cis-acting replication element in its genomic RNA (37) and SARS CoV-2 replication by interacting with the viral helicase NSP13 (38). However, its role in alphavirus replication is not known. We therefore further studied the EWSR1 binding to the CHIKV RNA and its role in virus replication in HEK293T cells.

### EWSR1 shows binding to 3NCR48 and 5NCR-RC50 sequences

**Fig 3:**
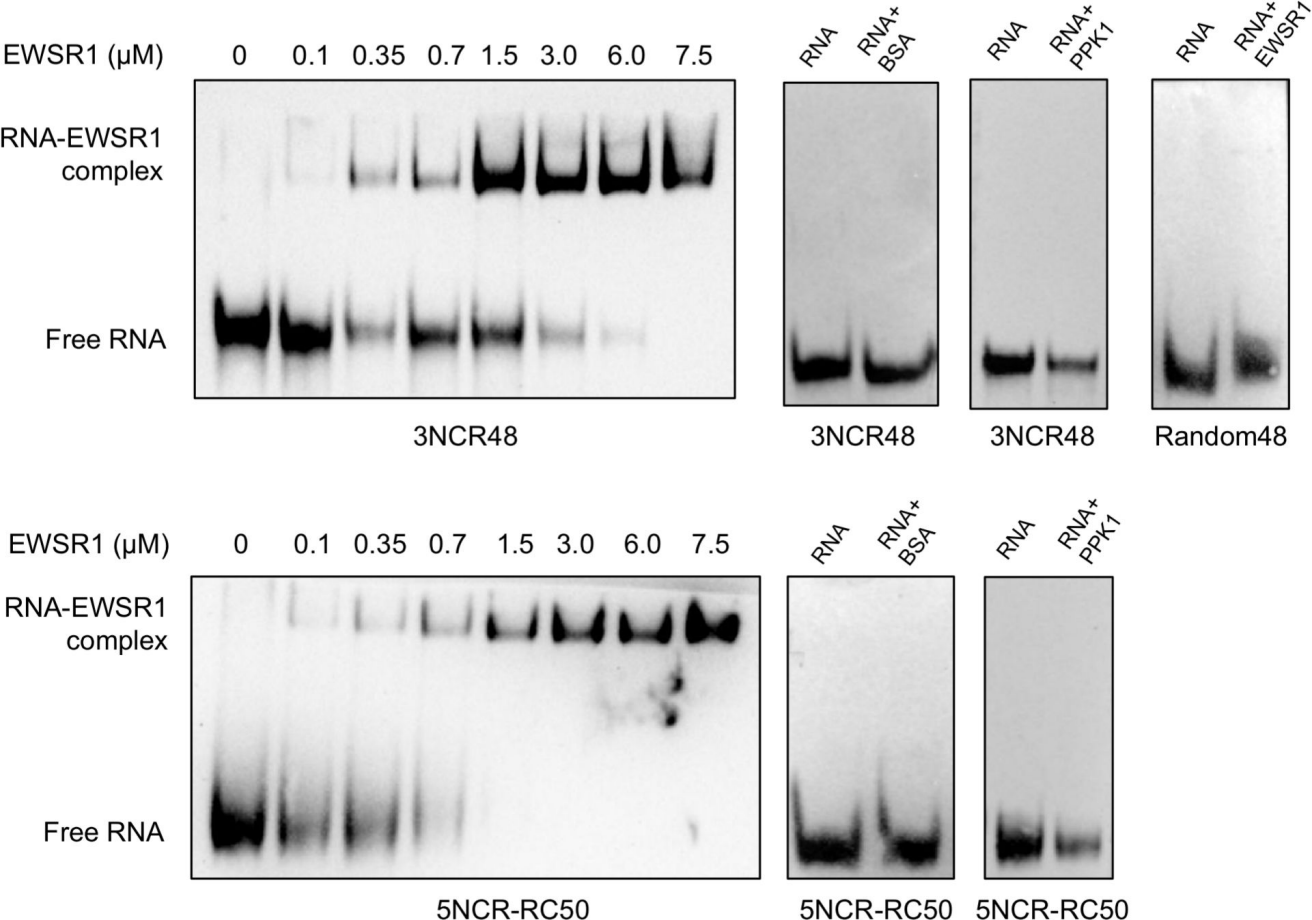
EWSR1 binding to CHIKV RNA. EMSA was used to study the binding of EWSR1 to 3NCR48 and 5NCR-RC50. Biotin-labeled synthetic RNA (10 ng) was incubated in the binding buffer with the indicated amounts of purified protein in the presence of 10 μg polyIC. The RNA–protein complexes were electrophoresed on a nondenaturing 12% polyacrylamide gel and transferred to a membrane. The RNA and the RNA–protein complexes were visualized using a chemiluminescent nucleic acid detection method. BSA and PPK1 (10 μg each) and Random48 (10 ng) were used as the nonspecific protein and RNA controls, respectively.

Recombinant EWSR1 produced in *E. coli* was used to study its binding to 3NCR48 and 5NCR-RC50 sequences by EMSA. RNA–protein complexes were seen when EWSR1 was incubated with 3NCR48 or 5NCR-RC50 RNAs (Fig. 3). These RNAs bound specifically to EWSR1, as no RNA–protein complex was seen with BSA used as a negative control. The recombinant EWSR1 used in these assays has a histidine tag. To rule out the role of the histidine tag in RNA binding, EMSA was done using an unrelated, histidine-tagged mycobacterial protein, polyphosphate kinase 1 (PPK1), produced in *E. coli*. PPK1 showed no binding to 3NCR48 or 5NCR-RC50. The RNA–protein binding specificity was further established as EWSR1 showed no binding with a Random48, a 48-nucleotide nonspecific RNA with a randomized sequence derived from 3NCR48.

**Fig 4:**
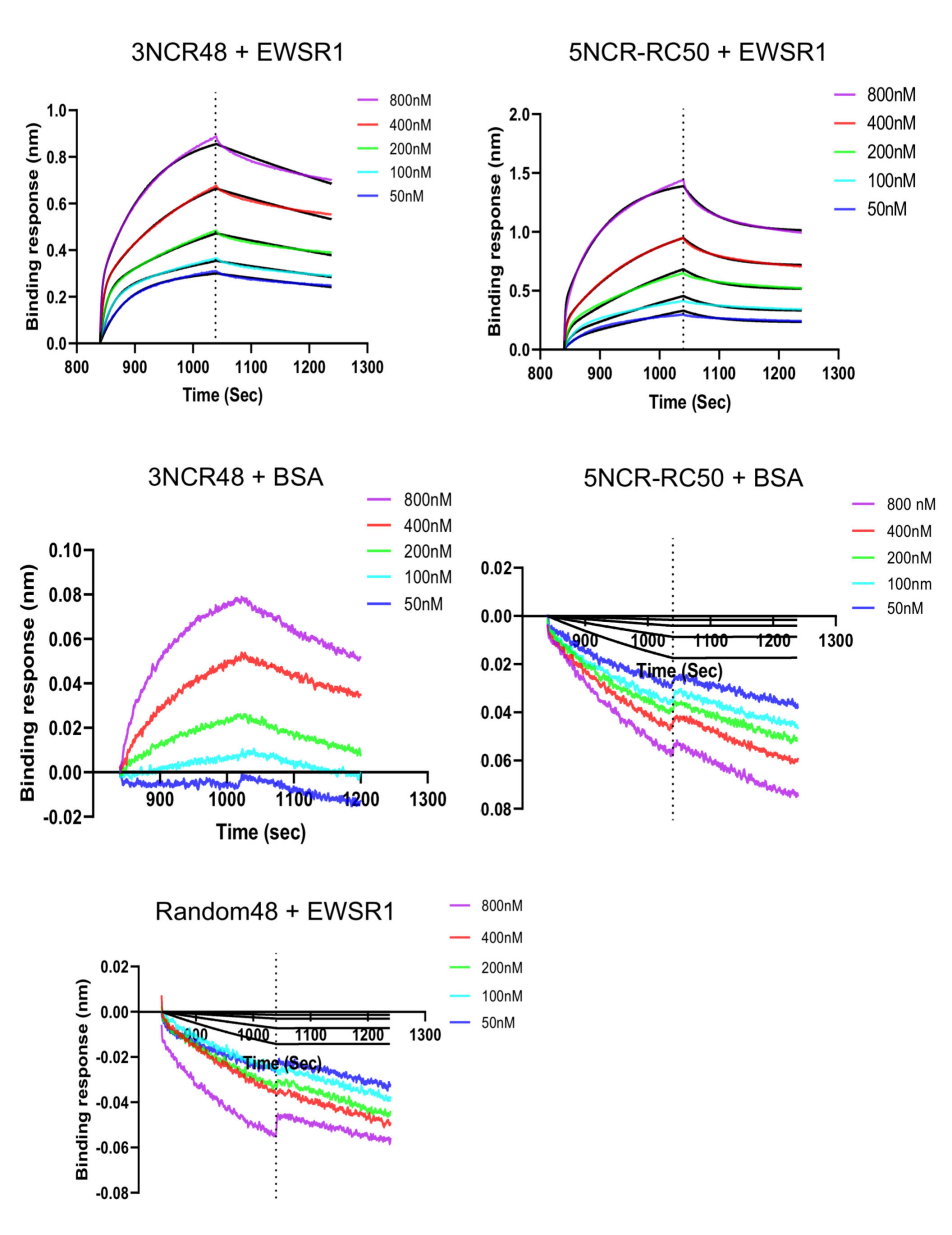
EWSR1 binding to CHIKV RNA. Biotinylated CHIKV RNAs 3NCR48 and 5NCR-RC50 (50 ng) were immobilized on the streptavidin-coated biosensor tips and exposed to increasing concentrations of EWSR1. Representative BLI binding curves showing association and dissociation are presented. BSA and Random48 were used as the nonspecific protein and RNA controls, respectively.

The RNA–protein binding was further studied using the label-free biolayer interferometry (BLI) method, where biotinylated CHIKV RNAs were immobilized on a streptavidin-coated sensor and then exposed to different concentrations of EWSR1 (Fig. 4). The protein bound to both 3NCR48 and 5NCR-RC50 RNAs with high affinity. The equilibrium dissociation constant (*K*_d_) was 91.4 ± 0.37 nM and 80.2 ± 0.39 nM for the EWSR1 binding to 3NCR48 and 5NCR-RC50 RNAs, respectively. The nanomolar *K*_d_ values suggest that these RNA–protein interactions may be physiologically plausible. In these experiments also, 3NCR48 and 5NCR-RC50 RNAs showed insignificant or no binding with the control protein BSA, and EWSR1 did not bind the control RNA, Random48. These data were consistent with the EMSA observations above.

### EWSR1 binds to CHIKV RNA in virus-infected cells

**Fig 5:**
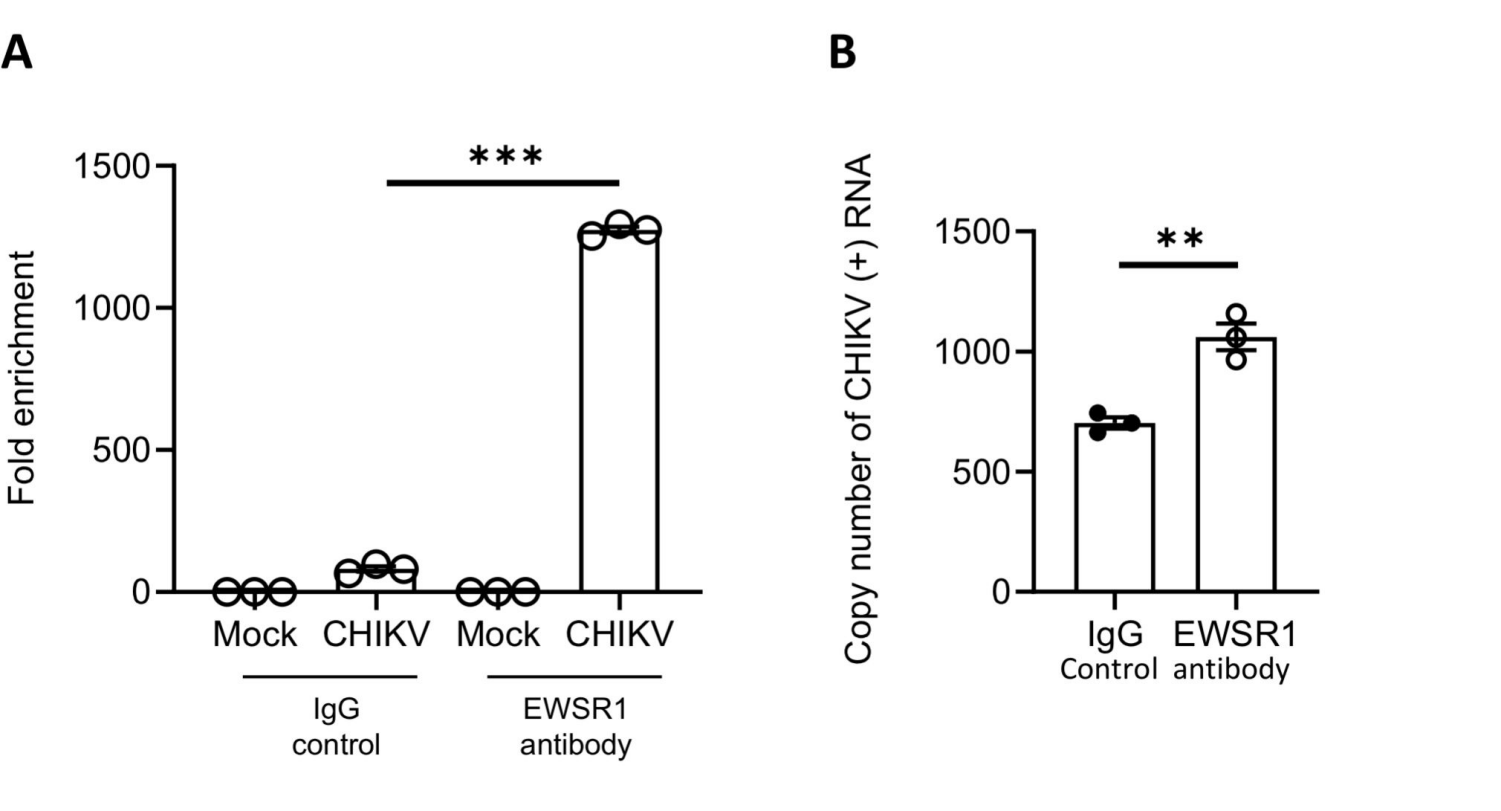
EWSR1 binds to CHIKV RNA in virus-infected cells. HEK293T cells were infected with CHIKV (MOI 0.5) or were mock-infected, and cell lysates were made at 12 hpi. Cell lysates from an equal number of cells were subjected to immunoprecipitation using anti-EWSR1 antibody or an IgG isotype control. (**A**) The co-immunoprecipitated RNA was extracted and analyzed by qRT-PCR using CHIKV-specific primers. Ct values were normalized to GAPDH, and relative fold changes were calculated with respect to the IgG control for the mock-infected cells. (**B**) Absolute copy numbers of CHIKV plus-sense RNA in the co-immunoprecipitated samples are presented.

RNA immunoprecipitation was employed to examine whether EWSR1 indeed bound CHIKV RNA during virus infection. Cell lysate from CHIKV-infected HEK293T cells was incubated with the EWSR1 antibody or the matching isotype IgG control, and the antibody–protein complex was pulled down. The complex was used to extract RNA, and qRT-PCR was done to determine the relative level of CHIKV RNA (Fig. 5A). A significantly increased (*P* < 0.001) amount of CHIKV RNA was detected in the complex pulled down by the EWSR1 antibody. We also determined the copy number of CHIKV plus-sense RNA associated with the pulled-down complex (Fig. 5B). Here also, significantly higher CHIKV RNA copies were pulled down by the EWSR1 antibody (*P* < 0.01). These data suggest that EWSR1 was associated with CHIKV RNA in the virus-infected HEK293T cells.

### CHIKV infection modulates EWSR1 expression

**Fig 6:**
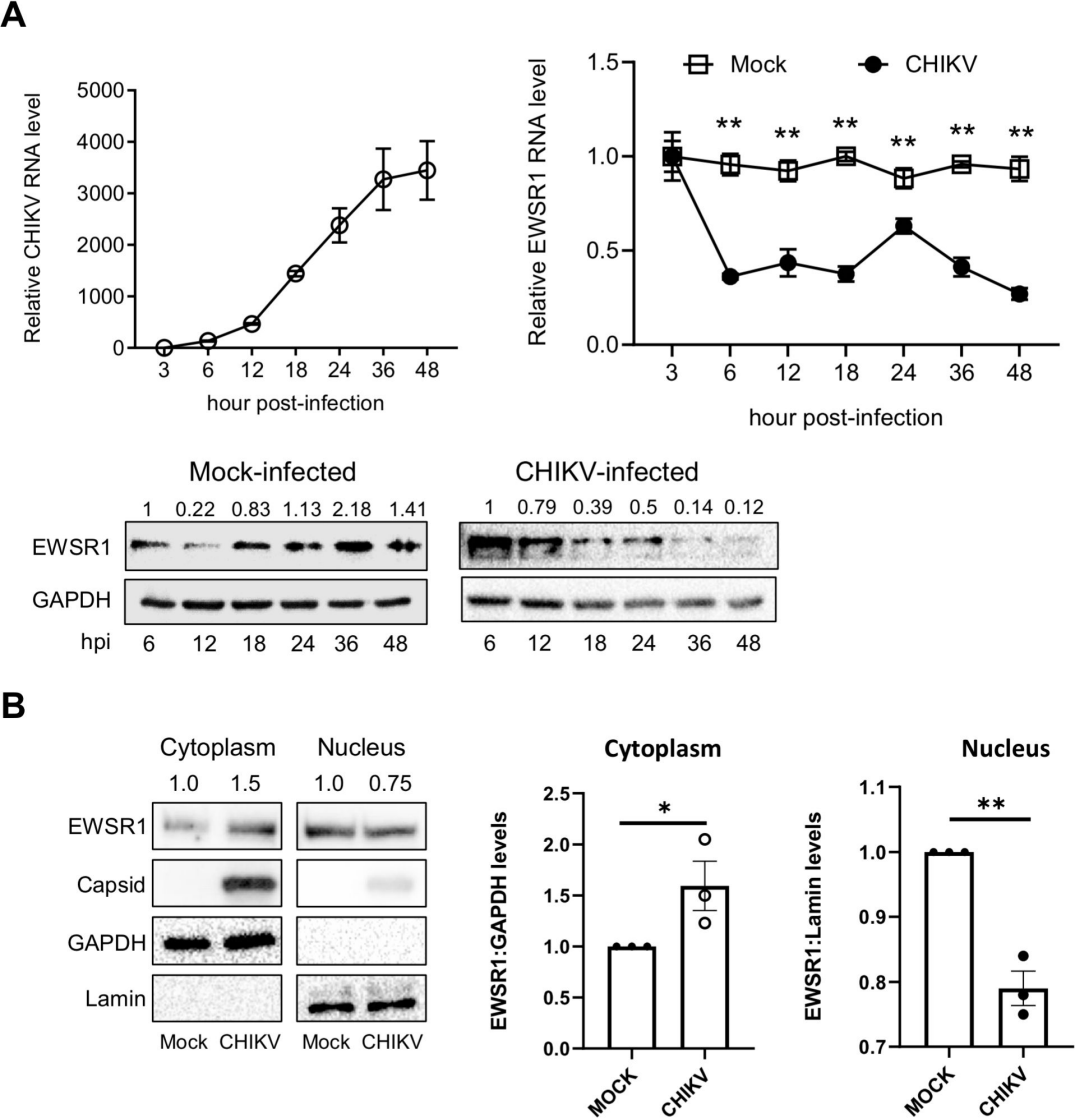
CHIKV infection modulates EWSR1 expression. (**A**) HEK293T cells were mock-infected or infected with CHIKV (MOI 0.5). The cells and culture supernatants were harvested at different times pi. CHIKV titers are shown in the top left panel. Cell lysates were used to extract total RNA, and the EWSR1 RNA levels in mock- and virus-infected cells were determined by qRT-PCR. GAPDH was used as an internal control for normalization. The experiment was performed in three biological replicates with three technical replicates for each. EWSR1 RNA levels, relative to those seen at 3 hpi in mock-infected cells, are presented in the top-right panel. Cell lysates were western blotted to determine EWSR1 protein levels in mock- and virus-infected cells. GAPDH RNA levels were used for normalization. The intensity of EWSR1 bands relative to GAPDH is shown at the top, where the EWSR1/GAPDH ratio at 6 hpi was taken as one. A representative western blot is shown. (**B**) HEK293T cells were mock-infected or infected with CHIKV (MOI 0.5). Cells were harvested at 12 hpi, and the cell lysates were separated into nuclear and cytoplasmic fractions. GAPDH and Lamin were blotted to establish the purity of the cytoplasmic and nuclear fractions, respectively. Western blots from a representative experiment are shown at the left, where the relative levels of EWSR1 protein compared to GAPDH (in cytoplasm) and Lamin (in nucleus), as measured by band intensities, are shown at the top. The relative value in the mock-infected cells was taken as one. The graphs at the right show relative EWSR1 protein levels in the nucleus and cytoplasm from three independent experiments.

The effect of CHIKV infection on EWSR1 expression was studied in HEK293T cells by determining the EWSR1 RNA levels by qRT-PCR and protein levels by western blotting. As the infection progressed, as determined by CHIKV RNA levels in cells (Fig. 6A, top left panel), the EWSR1 RNA levels dropped significantly early during the infection (3–6 hpi), after which not much change was recorded (Fig. 6A, top right panel). The virus infection led to reduced EWSR1 protein levels (Fig. 6A, bottom panel). While reduced EWSR1 RNA levels might have contributed to the reduced EWSR1 protein levels, it may also be a result of the global host protein synthesis shutdown during alphavirus replication (39–41).

EWSR1 is a protein primarily localized within the nucleus, where it plays important roles in transcriptional regulation and RNA processing (42). It has also been found in the cytoplasm; its subcellular localization is affected by the methylation process (43). Several RBPs show nucleus-to-cytoplasm shuttling during RNA virus infection. For example, nuclear RBPs such as MATR3 and DDX21 translocate to the cytoplasm upon SINV or Semliki Forest virus (SFV) infection to interact with viral RNA (44). We have previously shown cytoplasmic translocation of polypyrimidine tract-binding (PTB) protein and its binding to viral RNA during Japanese encephalitis virus (JEV) infection (45). CHIKV replicates in the cytoplasm (46), and we have shown that EWSR1 interacts with CHIKV RNA. In this context, we studied the localization of EWSR1 in CHIKV-infected HEK293T cells by Western blotting of the nuclear and cytoplasmic fractions. In the mock-infected cells, EWSR1 was found in the nucleus as well as the cytoplasm. However, during CHIKV infection, the EWSR1 levels in the nucleus were reduced by ~20% (*P* < 0.01) and its cytoplasmic levels increased by ~60% (*P* < 0.05) (Fig. 6B). Together, these data showed that CHIKV infection downregulated the expression of EWSR1 RNA and protein and resulted in EWSR1 protein accumulation in the cytoplasm in HEK293T cells, where CHIKV replication takes place.

### EWSR1 has an antiviral role in CHIKV replication

**Fig 7:**
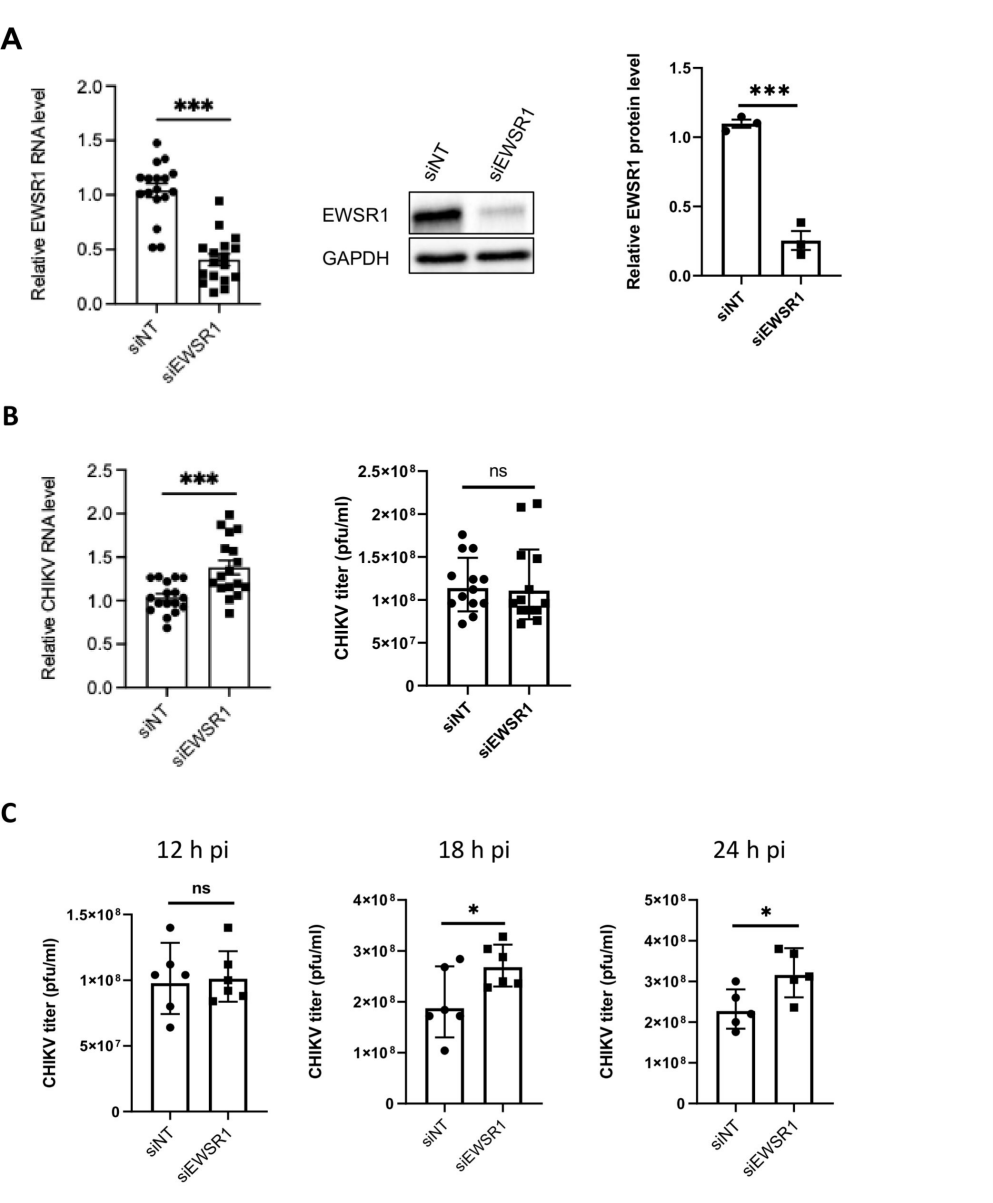
CHIKV replication in the siRNA-mediated EWSR1-downregulated cells. (**A**) HEK293T cells were transfected with siEWSR1 or a control nontargeting siNT and harvested 48 h later to determine the relative EWSR1 RNA and protein levels by qRT-PCR and western blotting, respectively. The left panel shows the relative EWSR1 RNA levels compared to those in the siNT-treated cells. The right panel shows the relative EWSR1 protein levels compared to those in the siNT-treated cells, based on the band intensity in the western blots. A representative western blot is shown in the middle. (**B**) HEK293T cells were transfected with siEWSR1 or siNT and 48 h later infected with CHIKV (MOI 0.5). The cells and culture supernatants were harvested 12 h later. The CHIKV RNA levels in siEWSR1-treated cells relative to the siNT-treated cells are shown in the left panel. The GAPDH RNA levels were used as the internal control for normalization. CHIKV titers (geometric mean ± SD) are shown in the right panel. (**C**) HEK293T cells were transfected with siEWSR1 or siNT and 48 h later infected with CHIKV (MOI 0.5). The culture supernatants were harvested at the indicated time points. CHIKV titers (geometric mean ± SD) in the siNT- and siEWSR1-treated cells are shown. All experiments were performed in three biological replicates with six technical replicates for each.

siRNA-mediated knockdown of EWSR1 was used to study its role in CHIKV replication. HEK293T cells were treated with siEWSR1 or siNT (nontargeting control) for 48 h. This resulted in a ~60% reduction in the EWSR1 mRNA levels and a ~75% reduction in the protein levels (Fig. 7A). At this point, the cells were infected with CHIKV, and the cells and supernatant were harvested at different hpi to determine the viral RNA and titer, respectively. While a 35% enhancement of CHIKV RNA levels was seen at 12 hpi, the virus titer was unaffected in the siEWSR1-treated cells at this time point (Fig. 7B). In another experiment, CHIKV titers were measured at later time points of 18 and 24 hpi (Fig. 7C). Here also, viral titers were not affected at 12 hpi, although they were significantly lower (*P* < 0.05) by 30%–40% at 18 and 24 hpi in the siEWSR1-treated cells than in the control.

**Fig 8:**
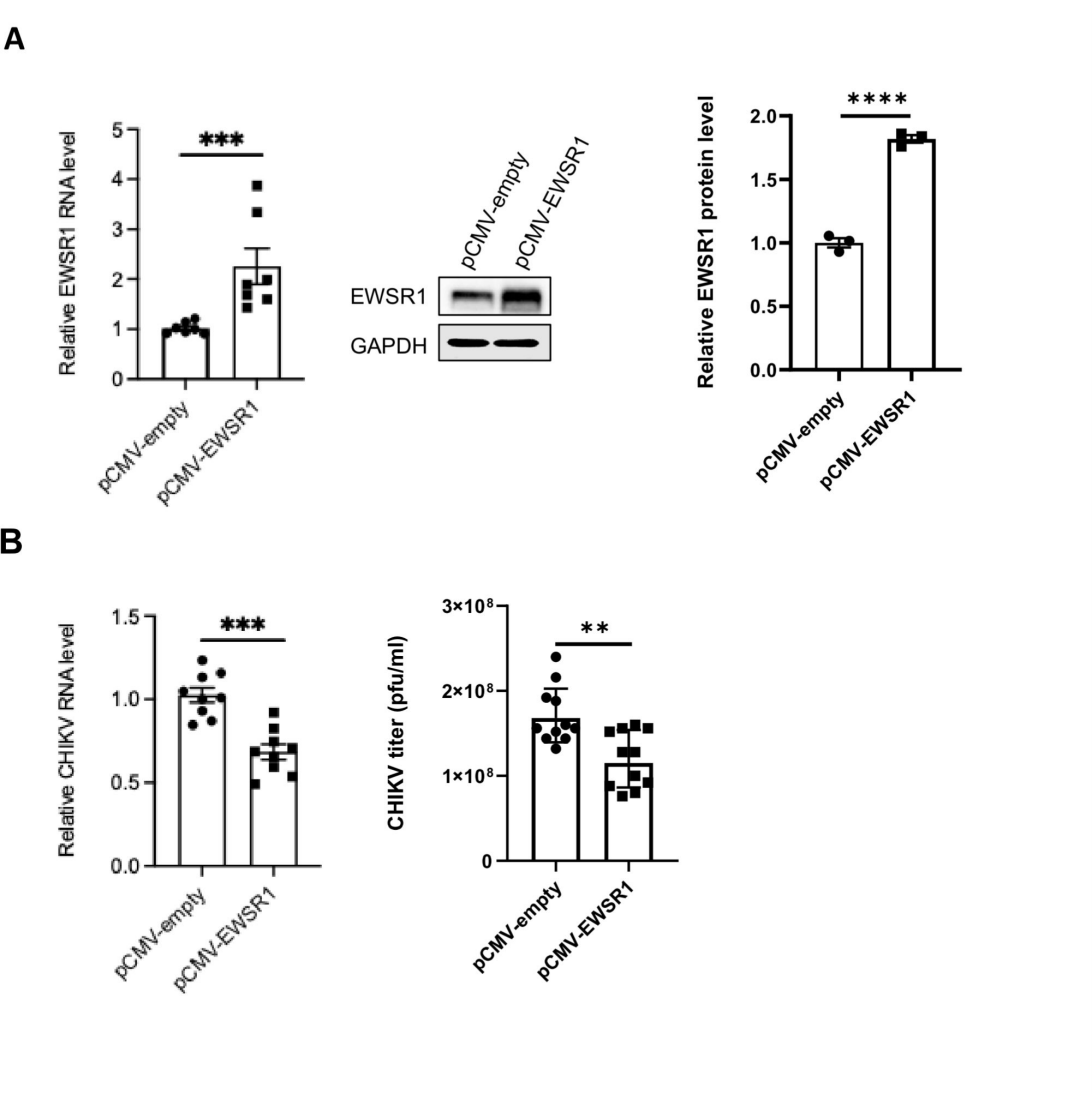
CHIKV replication in ectopically EWSR1 expressing cells. (**A**) HEK293T cells were transfected with the empty vector pCMV-empty or EWSR1-expressing pCMV-EWSR1 and harvested 24 h later to determine the relative EWSR1 RNA and protein levels by qRT-PCR and western blotting, respectively. The left panel shows the relative EWSR1 RNA levels compared to those in the pCMV-empty-transfected cells. The right panel shows the relative EWSR1 protein levels compared to those in the pCMV-empty-transfected cells. A representative western blot is shown in the middle. (**B**) HEK293T cells were transfected with pCMV-empty or pCMV-EWSR1, and 24 h later infected with CHIKV (MOI 0.5). The cells and culture supernatants were harvested 12 h later. CHIKV RNA levels in pCMV-EWSR1-transfected cells relative to pCMV-empty-transfected cells are shown in the left panel. The GAPDH RNA levels were used as the internal control for normalization. CHIKV titers (geometric mean ± SD) are shown in the right panel. All experiments were performed in three biological replicates with three technical replicates for each.

We also studied the effect of EWSR1 overexpression on CHIKV replication. HEK293T cells were transfected with a plasmid ectopically expressing EWSR1, which resulted in a 120% increase in the EWSR1 RNA levels and a 95% increase in EWSR1 protein levels at 24 h post-transfection (Fig. 8A). At this point, the cells were infected with CHIKV, and at 12 hpi, the samples were harvested to determine the CHIKV RNA and the virus titers. A significant decrease in CHIKV RNA by 35% (*P* < 0.001) and titers by 40% (*P* < 0.0.01) was seen in EWSR1-overexpressing cells (Fig. 8B).

**Fig 9:**
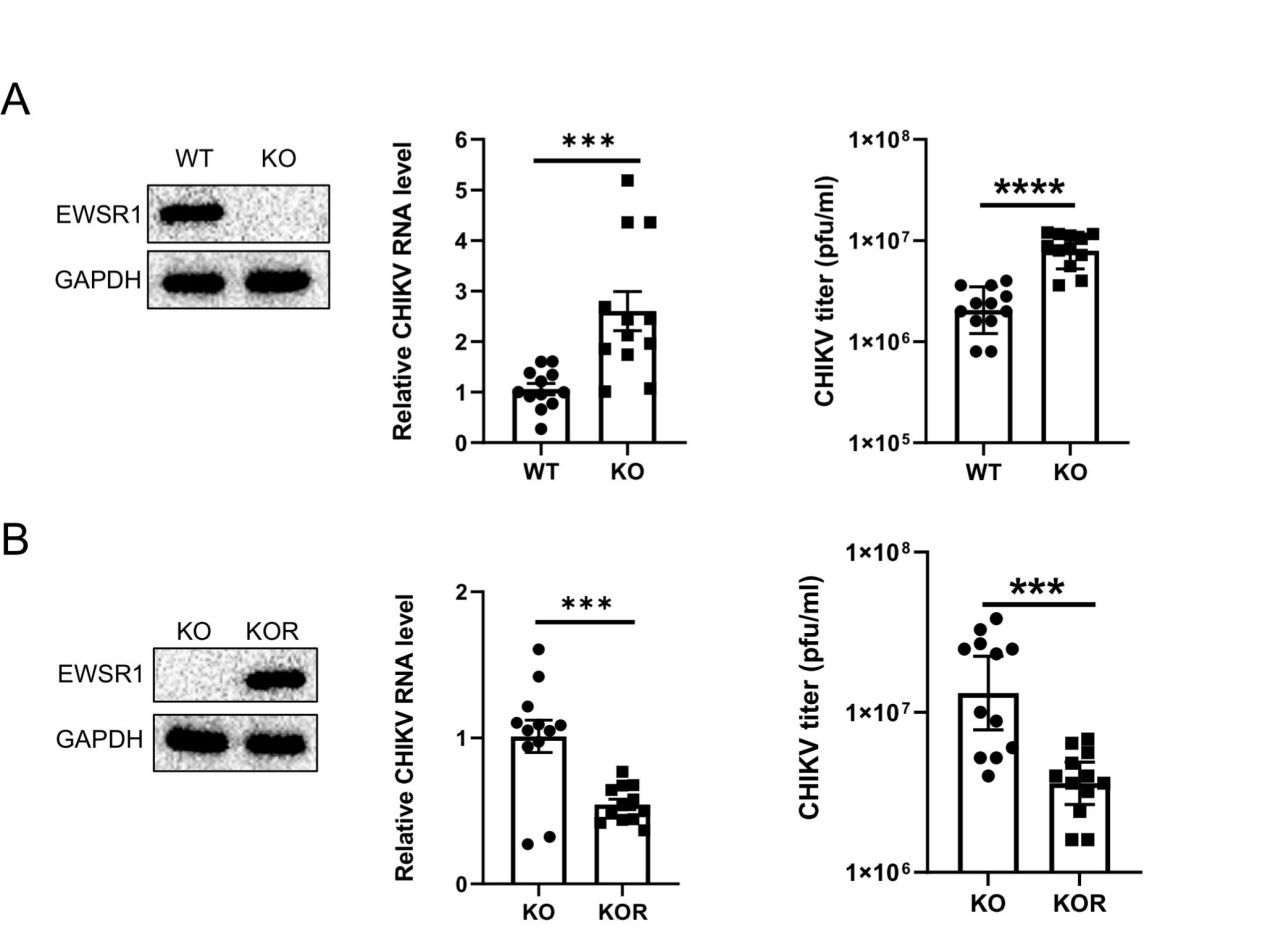
CHIKV replication in EWSR1 knockout HAP1 cells. (**A**) WT and EWSR1 KO HAP1 cells were cultured and Western blotted to establish the absence of EWSR1 protein in the KO cells (left panel). WT and KO HAP1 cells were infected with CHIKV (MOI 0.5), and 12 h later, CHIKV RNA levels in the KO cells relative to those in the WT cells were determined by qRT-PCR (middle panel). The GAPDH RNA levels were used as the internal control for normalization. The CHIKV titers (geometric mean ± SD) in the WT and KO cells are presented in the right panel. (**B**) EWSR1 KO HAP1 cells were transfected with an EWSR1-expressing plasmid pCMV-EWSR1. EWSR1 KO and EWSR1-rescued cells (KOR) were Western blotted 24 h later using the EWSR1 antibody to establish the presence of the ectopically expressed EWSR1 (left panel). KO and KOR cells were infected with CHIKV (MOI 0.5), and the cells and culture supernatants were harvested 12 h later. The viral RNA levels in the KOR cells relative to the KO cells are shown (middle panel). The GAPDH RNA levels were used as the internal control for normalization. The viral titers (geometric mean ± SD) are shown in the right panel. All experiments were performed in three biological replicates with four technical replicates for each.

To validate these findings and to rule out any off-target effects of siEWSR1, an EWSR1 KO of HAP1 cells was made. KO cells were infected with CHIKV, and at 12 hpi, the cells and supernatant were collected to determine viral RNA levels and titers. The CHIKV RNA levels and titers were significantly increased by 150% (*P* < 0.001) and 271% (*P* < 0.0.0001), respectively, in the KO cells compared to the wild-type (WT) control (Fig. 9A). We also rescued the EWSR1 expression in the EWSR1 KO HAP1 cells by transfecting them with a plasmid ectopically expressing EWSR1. In the EWSR1-rescued KO cells (KOR), CHIKV RNA levels were reduced by 45% (*P* < 0.001) and virus titers by 77% (*P* < 0.001), compared to the EWSR1 KO cells (Fig. 9B).

Together, these data demonstrate an antiviral role of EWSR1 in CHIKV replication.

### EWSR1 reduces both plus- and minus-sense CHIKV RNA levels in virus-infected cells

**Fig 10:**
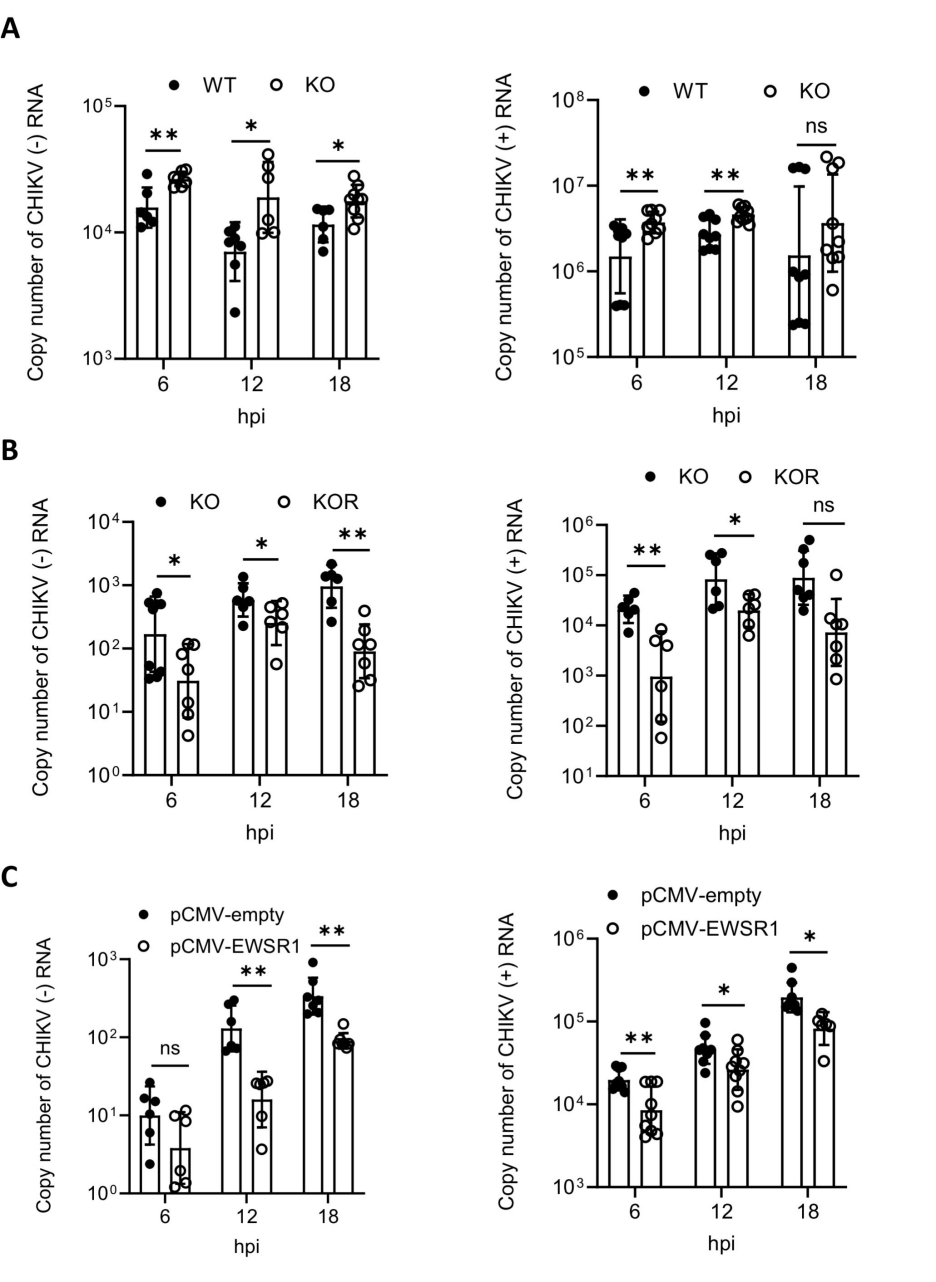
CHIKV plus- and minus-sense RNA copy numbers in virus-infected cells. (**A**) The EWSR1 WT and KO HAP1 cells were infected with CHIKV (MOI 0.5), and the cells were harvested at different hpi to isolate the total RNA. qRT-PCR was performed to determine the CHIKV plus- and minus-sense RNA copy numbers. (**B**) The EWSR1 KO and the KOR HAP1 cells were infected with CHIKV (MOI 0.5), and the cells were harvested at different hpi to isolate the total RNA. qRT-PCR was performed to determine the CHIKV plus- and minus-sense RNA copy numbers. (**C**) HEK293T cells were transfected with the empty vector pCMV-empty or the EWSR1-expressing pCMV-EWSR1, and 24 h later infected with CHIKV (MOI 0.5). The cells were harvested at different hpi to isolate the total RNA. qRT-PCR was performed to determine the CHIKV plus- and minus-sense RNA copy numbers. All experiments were performed in three biological replicates with two technical replicates for each. RNA copy numbers are presented as geometric mean ± SD.

EWSR1 can bind both plus- and minus-sense CHIKV RNAs. How this binding might affect the synthesis of these RNAs during virus replication was studied in HAP1 KO and WT cells (Fig. 10A). In the HAP1 KO cells, both minus- and plus-sense CHIKV RNA copy numbers were significantly higher than those in the WT cells (*P* < 0.05 or 0.01 at different time points). Ectopic expression of EWSR1 in the KO cells (KOR) significantly reduced both the minus- and plus-sense CHIKV RNA copy numbers (*P* < 0.05 or 0.01 at different time points) (Fig. 10B). This was further validated by ectopic expression of EWSR1 in HEK293T cells, where cells overexpressing EWSR1 showed significantly reduced copy numbers for both the minus- and plus-sense CHIKV RNAs (*P* < 0.05 or 0.01 at different time points) (Fig. 10C). Since EWSR1 binds sequences within the plus- and minus-sense CHIKV RNAs, this might have a role in the reduced viral RNA synthesis resulting in the lowering of CHIKV titers.

### CHIKV nsP2 binds 3NCR48 sequence

**Fig 11:**
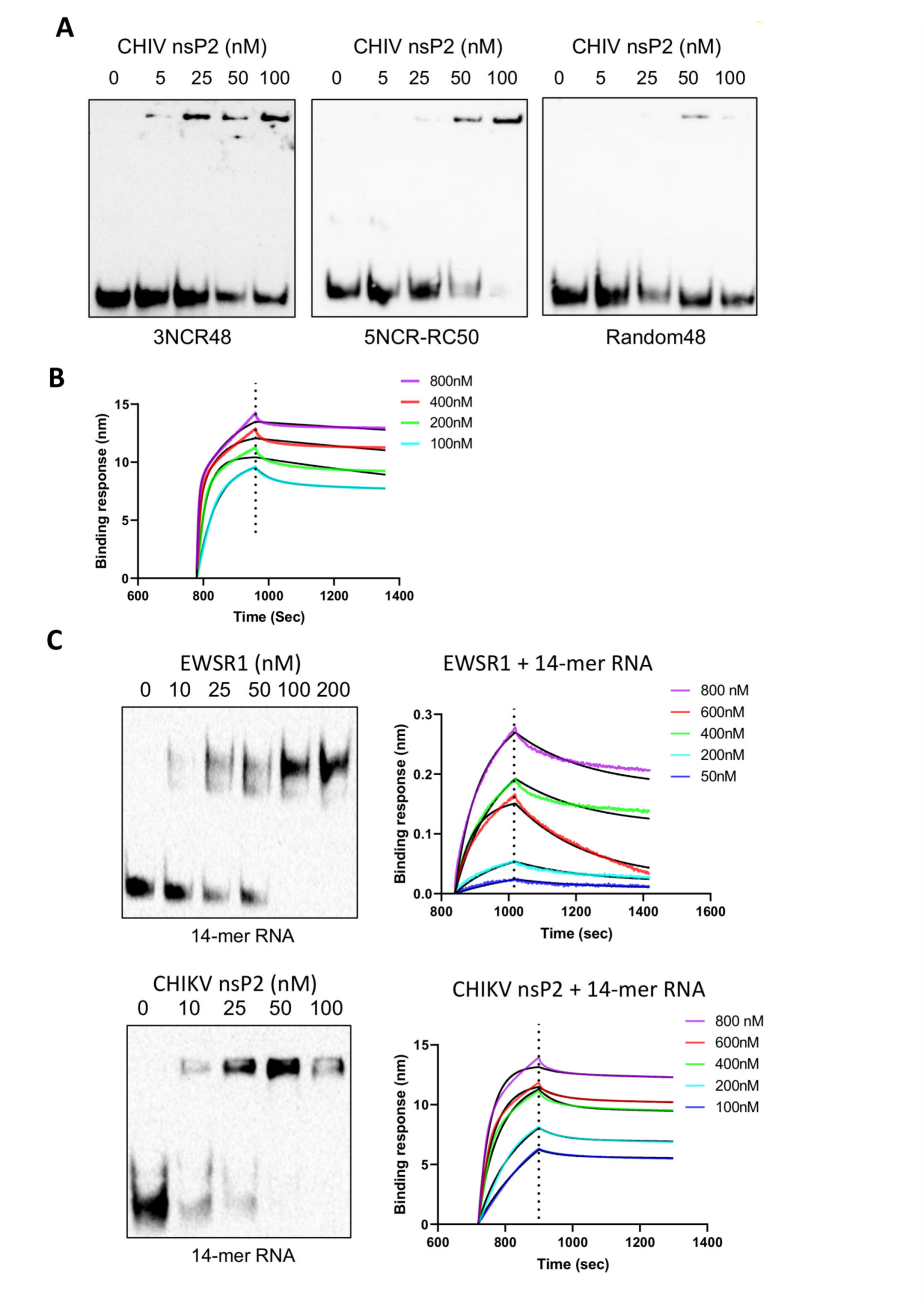
CHIKV nsP2 binds the 3NCR48 RNA. (**A**) EMSA was used to study the binding of CHIKV nsP2 to 3NCR48, 5NCR-RC50, and Random48 RNAs. Biotin-labeled synthetic RNA (20 ng) was incubated in the binding buffer with different amounts of CHIKV nsP2 protein in the presence of polyIC. The RNA–protein complexes were electrophoresed on a nondenaturing 12% polyacrylamide gel and transferred to the membrane. The RNA and the RNA-protein complexes were visualized using a chemiluminescent nucleic acid detection method. (**B**) Biotinylated 3NCR48 RNA (50 ng) was immobilized on the streptavidin-coated biosensor tips and exposed to increasing concentrations of CHIKV nsP2. Representative BLI binding curves showing association and dissociation are presented. (**C**) EMSA was used to study the binding of EWSR1 and CHIKV nsP2 with 14-mer RNA GUUUUUAAUAUUUC. Biotin-labeled synthetic RNA (20 ng) was incubated in the binding buffer with different amounts of protein in the presence of polyIC. The RNA–protein complexes were electrophoresed on a nondenaturing 12% polyacrylamide gel and transferred to the membrane. The RNA and the RNA-protein complexes were visualized using a chemiluminescent nucleic acid detection method. For the BLI studies, biotinylated RNA (50 ng) was immobilized on the streptavidin-coated biosensor tips and exposed to increasing concentrations of protein. Representative EMSA blots are shown on the left, whereas BLI binding curves showing association and dissociation are presented at the right.

EWSR1 binds 3NCR48 and 5NCR-RC50 sequences, which are at the extreme 3′-end of the CHIKV plus-sense genomic RNA and minus-sense replication-intermediate RNA, respectively. These ends interact with alphavirus nonstructural proteins to initiate RNA synthesis (11). In fact, the nucleotide sequence GUUUUUAAUAUUUC, which is part of the 3NCR48 sequence at its 3′-end (Table S1), has been shown to bind the CHIKV nsP2 helicase (47). We therefore sought to examine whether CHIKV nsP2 binds the 3NCR48 and 5NCR-RC50 RNAs. Indeed, EMSA showed CHIKV nsP2 binding to these two sequences (Fig. 11A). The protein also showed binding to the nonspecific RNA Random48, as a helicase is expected to bind any RNA. We then studied the binding of 3NCR48 RNA with CHIKV nsP2 by BLI, which validated the CHIKV nsP2 binding with the 3NCR48 RNA (Fig. 11B). The equilibrium dissociation constant (*K*_d_) for CHIKV nsP2 binding with 3NCR48 RNA was 92.9 ± 1.6 nM.

EWSR1 is known to bind the G/U- and A/U-rich sequences (48). A 14-mer G/U- and A/U-rich sequence GUUUUUAAUAUUUC is located at the 3′-end of 3NCR48 (Table S1). This sequence was also shown to bind CHIKV nsP2 (47). We, therefore, examined the binding of EWSR1 and nsP2 to this sequence (Fig. 11C). EMSA and BLI data show that this sequence indeed could bind both nsP2 as well as EWSR1.

### EWSR1 binds to CHIK nsP2

**Fig 12:**
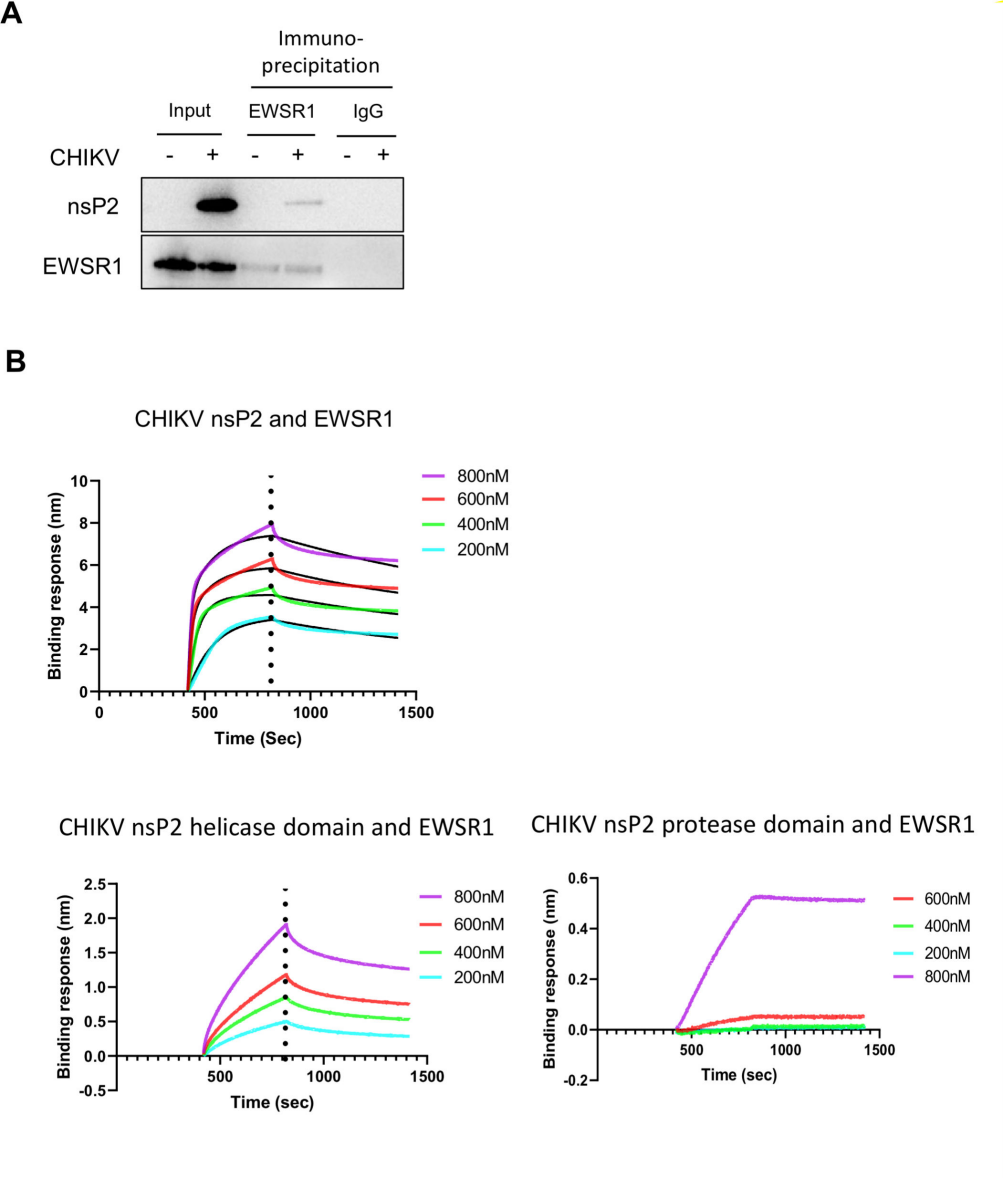
EWSR1 binds to CHIKV nsP2. (**A**) HEK293T cells were mock-infected (−) or infected (+) with CHIKV (MOI 0.5), and 12 h later, cell lysates (input) were prepared, which were subjected to immunoprecipitation using the EWSR1 antibody or rabbit IgG (isotype control). The immunoprecipitate was western blotted with anti-EWSR1 and anti-CHIKV nsP2 antibodies. (**B**) His-tagged EWSR1 (10 µg) was immobilized on Ni-NTA biosensor tips and exposed to increasing concentrations of CHIKV nsP2 protein, or its helicase and protease domain proteins. Representative BLI binding curves showing association and dissociation of the proteins at different nsP2 concentrations are presented.

In a recent study, EWSR1 was shown to interact with the SARS-CoV-2 helicase protein (38). We, therefore, examined whether EWSR1 was interacting with the CHIKV nsP2 protein, which has helicase activity. Using the CHIKV-infected HEK293T cell lysate, we immunoprecipitated EWSR1-interacting protein/s, if any, using the EWSR1 antibody. Interestingly, we detected CHIKV nsP2 protein in the immunoprecipitate but not in the IgG and mock controls (Fig. 12A), suggesting that EWSR1 may be interacting with CHIKV nsP2 during virus infection.

BLI method was employed to validate the binding of EWSR1 to CHIKV nsP2 (Fig. 12B). Recombinant histidine-tagged EWSR1 made in *E. coli* was immobilized on a Ni-NTA-coated biosensor and then exposed to different concentrations of CHIKV nsP2 protein purified from the recombinant *E. coli*. The BLI sensorgram demonstrated the binding of EWSR1 with CHIKV nsP2; the equilibrium dissociation constant (*K*_d_) was 716.2 ± 1.76 nM. CHIKV nsP2 is a multifunctional protein having distinct helicase and protease domains (47). The BLI method showed EWSR1 binding with the nsP2 helicase domain. However, no EWSR1 binding was seen with the nsP2 protease domain (Fig. 12B).

### EWSR1 inhibits CHIKV nsP2 helicase activity but not the protease activity

**Fig 13:**
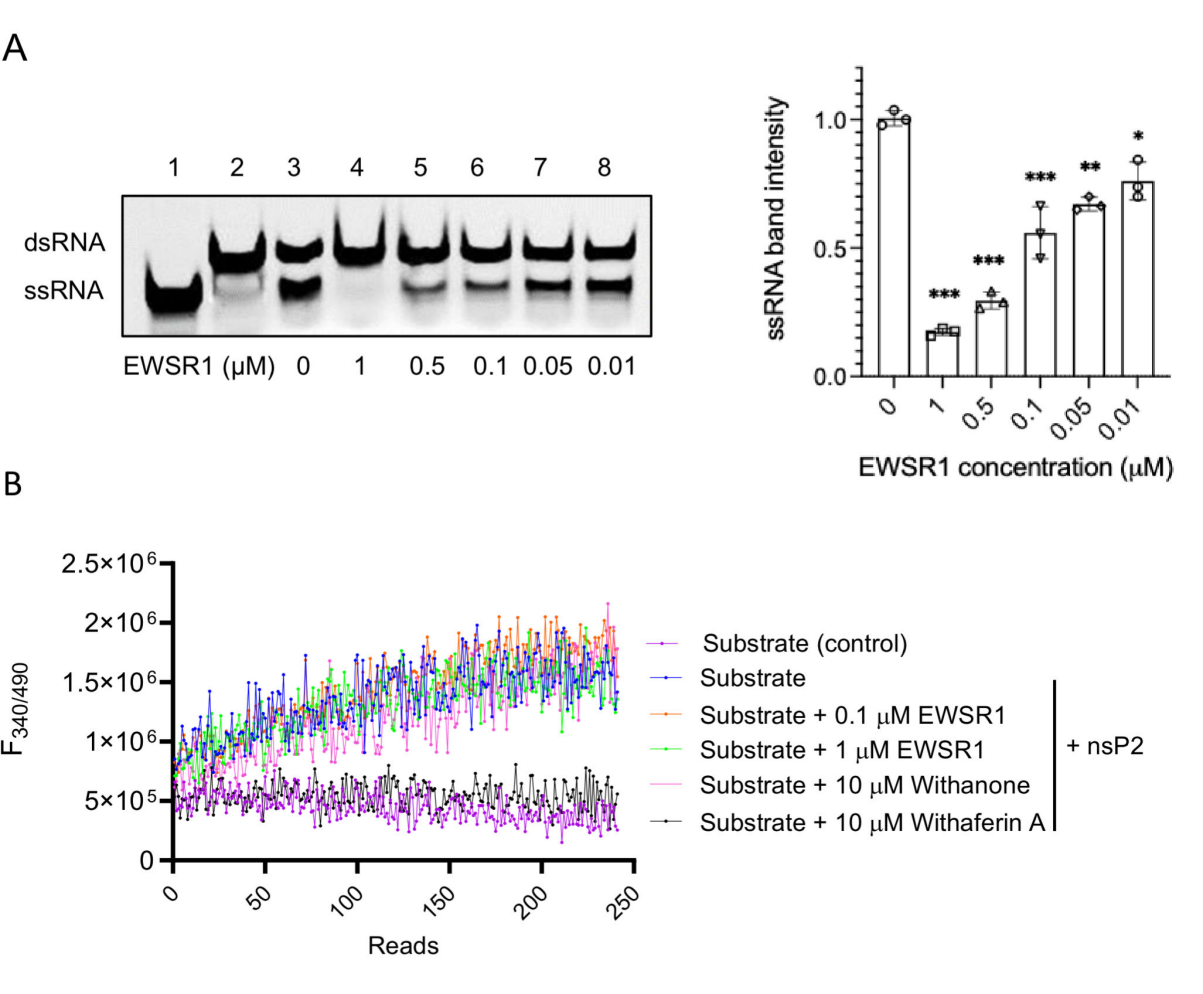
EWSR1 inhibits CHIKV nsP2 helicase activity. (**A**) The RNA helicase activity of CHIKV nsP2 in the presence of increasing EWSR1 concentrations was studied using Alexa488-dsRNA28/16 substrate. A representative gel picture of an experiment is shown in the left panel. Lane 1 has the substrate denatured by heat, thus producing ssRNA; lane 2 shows just the dsRNA substrate; lane 3 shows the helicase activity of CHIKV nsP2 in the absence of EWSR1 producing ssRNA; and lanes 4–8 show the nsP2 helicase activity inhibition in the presence of the indicated EWSR1 concentrations. (**B**) A FRET-based protease assay was used to study the nsP2 protease activity. A real-time profile of the proteolytic assay is presented using a 25 μM fluorogenic peptide substrate and 1 μM nsP2 protein, with different concentrations of EWSR1. Withaferin A, a known inhibitor of nsP2 protease, and withanone, known not to inhibit the nsP2 protease activity, were used as the controls. The fluorescence was monitored every 30 s. The background fluorescence of the substrate peptide was monitored without the enzyme and shown as a control (substrate). Data from a representative experiment are shown.

To examine whether the EWSR1 binding to CHIKV nsP2 affected its helicase activity, the nsP2 helicase assay was done in the presence of increasing amounts of EWSR1 (Fig. 13A). As expected, the CHIKV nsP2 protein was able to unwind the dsRNA substrate, producing ssRNA. The helicase activity of CHIKV nsP2 was inhibited by EWSR1 in a concentration-dependent manner. While partial inhibition of the helicase activity was seen at lower concentrations, a near-complete inhibition was seen in the presence of 1 μM EWSR1 protein.

A FRET-based assay has been established and validated in our lab to study CHIKV nsP2 protease activity (27). EWSR1 had no effect on the nsP2 protease activity when used at 1 μM concentration (Fig. 13B). Here, withaferin A, which has been shown to inhibit CHIKV nsP2 protease activity, was used as a positive control, and withanone, a known protease inhibitor, was used as a negative control for protease activity inhibition, and both controls worked as reported previously (27). The qualitative findings of the protease assay were confirmed upon repetition.

### Role of EWSR1 in the replication of other alphaviruses

**Fig 14:**
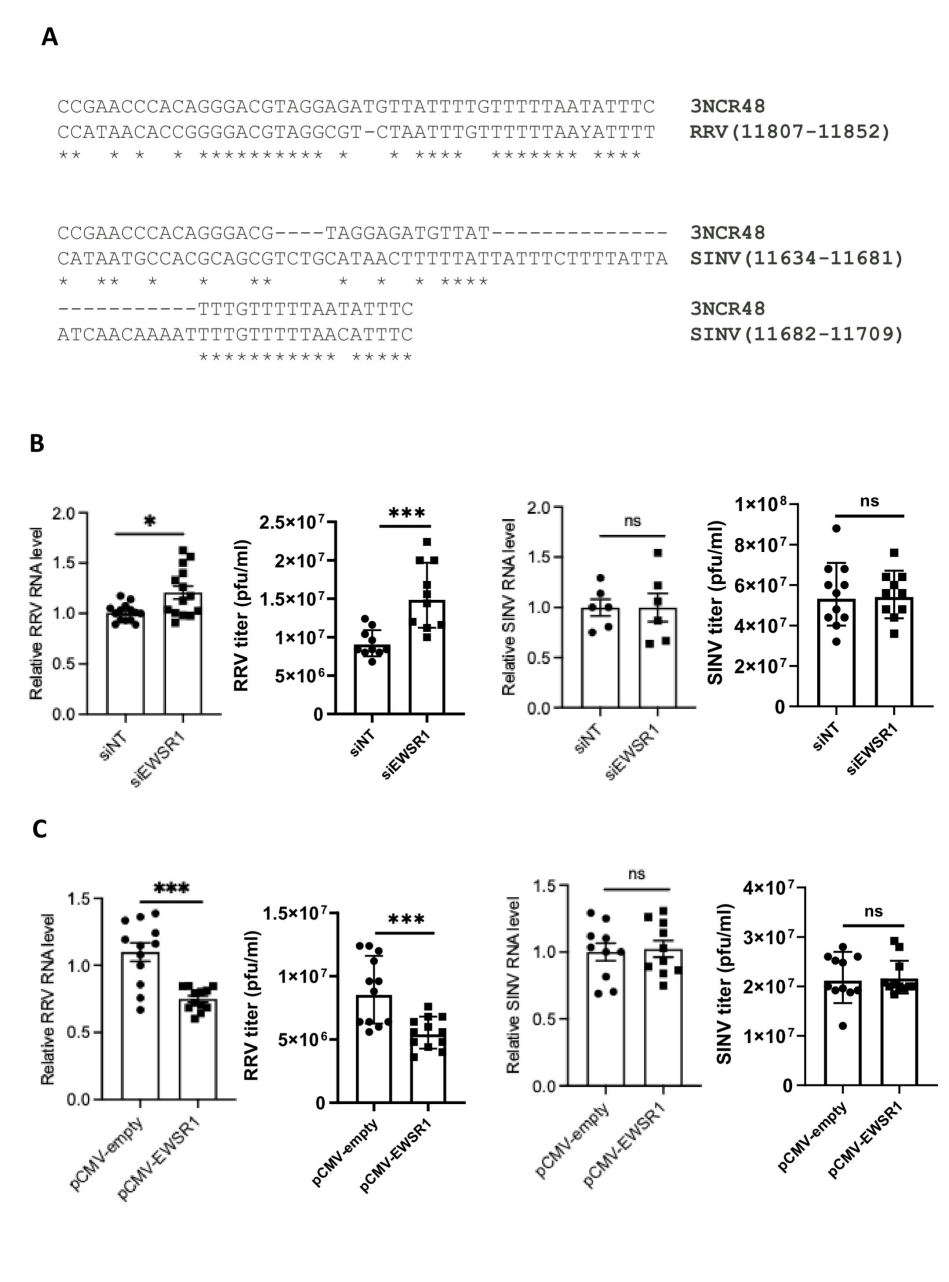
Role of EWSR1 in the replication of RRV and SINV. (**A**) CHIKV 3NCR48 sequence was aligned with the RRV (GenBank ID GQ433359.1) and SINV (GenBank ID LC869668.1) RNA using Clustal Omega. Conserved nucleotides are identified with an asterisk underneath. The position of the aligned sequence on the RRV or SINV genome is identified by the nucleotide position in the brackets. (**B**) HEK293T cells were transfected with siEWSR1 or a nontargeting siNT and 24 h later infected with RRV or SINV (MOI 0.5). The cells and culture supernatants were harvested 12 h later. The viral RNA levels in siEWSR1-treated cells relative to the siNT-treated cells are shown. The GAPDH RNA levels were used as the internal control for normalization. Viral titers (geometric mean ± SD) are also shown. (**C**) HEK293T cells were transfected with pCMV-empty or pCMV-EWSR1 and 24 h later infected with RRV or SINV (MOI 0.5). The cells and culture supernatants were harvested 12 h later. The CHIKV RNA levels in pCMV-EWSR1-transfected cells relative to the pCMV-empty-transfected cells are shown. The GAPDH RNA levels were used as the internal control for normalization. Viral titers (geometric mean ± SD) are also shown. All experiments were performed in three biological replicates with six technical replicates for each.

Clustal Omega was used to align the CHIKV 3NCR48 sequence with the 3′-NCR sequences of SINV and RRV, and LALIGN was used to calculate the sequence conservation (Fig. 14A). The CHIKV 3NCR48 sequence showed 82.6% similarity to the RRV sequence, whereas only 51.3% similarity of this sequence was seen in SINV. In fact, a large insertion of 25 nucleotides was observed in the corresponding SINV sequence when compared with the CHIKV 3NCR48 sequence. We, therefore, chose these two alphaviruses to study the role of EWSR1 interaction with the 3NCR48 sequence in virus replication.

siRNA-mediated EWSR1-depleted HEK293T cells were infected with RRV or SINV to understand the role of EWSR1 in the replication of other alphaviruses (Fig. 14B). The viral RNA levels and titers were significantly higher (*P* < 0.05 and *P* < 0.001, respectively) in the siEWSR1-treated RRV-infected cells compared to the siNT-treated RRV-infected control cells. However, SINV RNA levels and virus titers remained unaffected in these cells. Replication of RRV and SINV was also studied in the EWSR1-overexpressing cells (Fig. 14C). While RRV RNA levels and titers were significantly lower (*P* < 0.001) in the cells ectopically overexpressing EWSR1, no effect on SINV RNA levels and titers was seen. These data thus demonstrate the importance of EWSR1 interaction with the 3NCR48 sequence to control virus replication.

## DISCUSSION

NCRs in the alphavirus genomes play an essential role in the viral replication process. The 5′- and 3′-NCRs contain critical structural and sequence elements that regulate both genome replication and translation (8). Host proteins specifically binding to these sequences are therefore likely to have a role in virus replication. Indeed, several host proteins have been shown to bind the CHIKV NCRs and play significant roles in viral replication. Musashi RBPs (MSI-1 and MSI-2) specifically bind to the 5′-NCR of CHIKV RNA. Depletion of these proteins blocks viral replication, showing a proviral role for them (34). Host cellular protein HuR binds alphavirus 3′-NCRs, including CHIKV, stabilizing viral RNA and preventing degradation, thus facilitating virus replication (18). Heterogeneous nuclear ribonucleoprotein K (hnRNP K) is an RBP that positively regulates CHIKV replication by modulating the subgenomic RNA (49).

We have identified several host proteins that bind specifically to the synthetic RNAs 3NCR48, 3NCR40, 5NCR51, and 5NCR-RC50, representing sequences located within the CHIKV NCRs or in the immediate vicinity, important for RNA replication. Conceivably, the functional annotation of these proteins showed that a majority of these proteins could bind nucleic acid, in particular RNA. The identified host proteins were broadly classified into RNA-binding hnRNPs, splicing regulators, mRNA stability factors, RNA 3′-end processing proteins, stress-granule–associated factors, and metabolic enzymes, highlighting extensive engagement of the host RNA metabolism pathways by the viral RNA. Importantly, different proteins bound to different RNAs, indicating the specificity of the RNA-protein binding. Interestingly, one protein, EWSR1, bound both the plus-sense (3NCR48 sequence) and minus-sense (5NCR-RC50 sequence) CHIKV RNAs in HEK293T cells, and therefore, we studied the role of this protein in further detail.

Some of the proteins identified here as binding to CHIKV RNA have been shown to bind to other viral RNAs and control virus replication. PSPC1 binds the HCV internal ribosome entry site (IRES) in the 5′-NCR, preventing ribosomal protein S5 recruitment and inhibiting translation initiation, with partial depletion enhancing viral replication (50). HNRDL (hnRNP D) also interacts with the HCV IRES in the 5′-NCR, functioning as an IRES trans-acting factor (ITAF) to boost IRES-driven translation, though depletion paradoxically increases replication by rebalancing translation and RNA synthesis (51). ELAV1 (HuR) binds AU-rich elements in the hepatitis B virus (HBV) pregenomic RNA (pgRNA), stabilizing it and facilitating its transport to the cytoplasm for replication. Knockdown of ELAV1 reduced pgRNA levels and suppressed HBV replication (52). PUF60 associates with HBV pgRNA, upregulates the core promoter while promoting pgRNA degradation and inhibiting splicing to favor virus replication (53). TIA1 binds to the uridine-rich regions of Tick-borne encephalitis virus (TBEV) RNA and inhibits translation via stress granule-like sequestration, with depletion boosting replication (54). TIA-1 plays a proviral role in West Nile virus (WNV) replication by binding to the 3′ stem-loop structure on the complementary minus-strand RNA, facilitating viral genome synthesis (55). In addition, G3BP1 and TIA1 participate in stress granule (SG) physiology, impacting virus replication by modulating SG assembly that sequesters viral RNA or activates antiviral signaling. G3BP1 is a core SG nucleator; its depletion impairs alphavirus (CHIKV and SINV) replication by blocking the translation-to-replication switch (56). TIA1 drives SG formation to inhibit flavivirus (TBEV and WNV) translation at replication sites, with depletion enhancing titers; the protein binds to U-rich viral RNAs to trap them in SGs (54).

The 3NCR48 sequence represents a 48-nucleotide conserved sequence within the alphavirus 3′-NCR. It is located immediately upstream of the poly(A) tail and functions as a *cis*-acting element that promotes efficient initiation of minus-strand RNA synthesis and supports optimal genome replication and stability (11, 57). Mutagenesis and infectious clone studies show that deletions or alterations in this 48-nucleotide conserved sequence markedly reduce minus-strand synthesis, plaque size, progeny production, and compromise infectivity in both vertebrate and invertebrate hosts, underscoring its essential, evolutionarily conserved role in the alphavirus life cycle (58). The 50-nucleotide 5NCR-RC50 sequence, which is complementary to the 5′-NCR of the virus genome, forms part of the core promoter on the negative-strand template that directs initiation of the plus-sense genomic RNA synthesis, thereby ensuring efficient genome replication during the alphavirus life cycle (59, 60). Mutational analyses show that this region acts as a replication enhancer whose sequence and predicted AU-rich unpaired structure are critical for the positive-strand synthesis in both mosquito and vertebrate cells during alphavirus replication (61). EWSR1, a host protein that binds to the 3NCR48 and 5NCR-RC50 sequences, which represent the genome sequence elements critical for alphavirus replication, is therefore likely to affect the viral life cycle.

EWSR1/EWS RNA-Binding Protein 1 is a ubiquitously expressed multifunctional protein. EWSR1 participates in transcriptional co-regulation and RNA-binding activities, influencing gene expression programs and RNA maturation pathways, including miRNA biogenesis through Drosha interaction. It plays roles in chromatin remodeling and epigenetic regulation that affect cellular differentiation and development. EWSR1 modulates mitochondrial biogenesis and energy homeostasis by controlling the stability of PGC-1α, a critical regulator of mitochondrial function. Mutations and fusion proteins involving EWSR1 have been linked to cancers and neurological diseases such as amyotrophic lateral sclerosis (ALS). EWSR1 fusion proteins drive certain sarcoma oncogenesis; however, functions outside cancer remain broad but less well-defined (36). Importantly, EWSR1 acts as a host factor for HCV by binding to a critical RNA element (CRE), modulating its activity, physically associating with viral replication sites, and thereby facilitating efficient viral RNA replication and production of infectious virus. This mechanism appears to be specific to replication rather than translation of viral proteins (37). In addition, EWSR1 acts as a host factor enhancing the replication of SARS-CoV-2 by binding to and promoting the helicase function of NSP13 (38). However, our study shows that binding of EWSR1 to CHIKV nsP2 negatively modulates its helicase activity and reduces CHIKV replication.

No consensus RNA sequence exists for EWSR1 binding, although the protein prefers G/U- and A/U-rich sequences (48). Several RanBP2-type zinc finger proteins have been shown to bind the AGGUAA sequence with varying affinity, and EWSR1 is one such protein, binding to the AGGUAA sequence with moderate affinity (62). Both 3NCR48 and 5NCR-RC50 sequences (Table S1) contain several GU or AU stretches, although the AGGUAA sequence was not found. In our study, a G/U and A/U rich 14-mer sequence GUUUUUAAUAUUUC located at the 3-end of 3NCR48 was shown to bind EWSR1. Further studies on EWSR1-binding RNAs, including the CHIKV RNAs, will help identify a consensus EWSR1-binding RNA motif.

Our studies show that EWSR1 binds both 3NCR48 and 5NCR-RC50 sequences, located in the plus- and minus-sense CHIKV genomic RNAs, respectively. Direct binding of these sequences to purified EWSR1 was shown by EMSA as well as BLI. The protein was also shown to bind to CHIKV RNA in virus-infected HEK293T cells. During CHIKV infection, the EWSR1 protein was shown to accumulate in the cytoplasm of HEK293T cells. This is consistent with the fact that alphavirus RNA replication takes place in the cytoplasm (63, 64). During HCV infection, EWSR1 was also shown to relocate to the cytoplasm (37). EWSR1 is functionally hijacked by binding to SARS-CoV-2 NSP13 helicase and is likely to be relocalized from the nucleus to the cytoplasm. However, the modulation of the EWSR1 protein level and its translocation during SARS-CoV-2 replication was not reported (38).

In the case of HCV as well as SARS-CoV-2, EWSR1 was a positive modulator of virus replication (37, 38). Our study showed that CHIKV replication was significantly enhanced in siRNA-mediated EWSR1-depleted HEK293T cells as well as in the EWSR1 KO HAP1 cells. In contrast, ectopic expression of EWSR1 in HEK293T cells or in the EWSR1 KO HAP1 cells significantly suppressed CHIKV replication. These data demonstrate an antiviral role for EWSR1 in CHIKV replication.

There are several examples of host proteins that bind the NCRs of RNA viruses and have a proviral role. We have previously shown that La protein binds to the 3′- and 5′-NCRs of the JEV genome to circularize it and enhance virus replication (65, 66). La protein has also been shown to bind to the 3′-NCR of HCV RNA and promote viral RNA stability and replication (67). Our previous work has shown that polypyrimidine tract-binding protein (PTB) interacts with both the 5′-NCR of the positive-sense genomic RNA of JEV and its complementary sequence in the negative-sense replication intermediate and inhibits virus replication (45). In contrast, PTB binding to the 5′- and 3′-NCRs of poliovirus and HCV enhances viral RNA translation and replication by facilitating RNA circularization (68). RPL17 and YBX1 proteins bind the HCV 3′-NCR to enhance production of infectious particles (69). NF90/NF110 binds viral 5′- and 3′-NCRs and stabilizes viral RNA, enhancing replication of dengue virus and HCV (70, 71). Heterogeneous nuclear ribonucleoprotein hnRNP A1 binds to the NCRs of various viruses including SINV, EV71, and HCV, facilitating viral RNA replication (16, 72). In the case of foot-and-mouth disease virus (FMDV), the poly(A)-binding protein (PABP) interacts with 3′-NCR and promotes translation initiation and viral RNA stability (73). Enolase (ENO), a host glycolytic enzyme, binds bamboo mosaic virus (BaMV) NCRs to enhance replication (74). DDX3 regulates the cap-independent translation of the JEV genome via its interactions with PABP1 and the 5′- and 3′-NCRs of the viral genome (75). We previously showed that nucleolin protein binds to the 3′-NCR of JEV and enhances virus replication (76).

Importantly, there are also many examples of host proteins binding the NCRs of RNA viruses showing antiviral activity. The 5′-NCR of alphaviruses, like CHIKV, contains conserved RNA structures and a 5′-ppp moiety that binds the IFIT1 protein, thereby blocking the assembly of the translation initiation complex. This inhibits viral protein synthesis, resulting in viral replication inhibition (77). For another alphavirus, Venezuelan equine encephalitis virus, the IFIT2 protein binds the 3′-NCR and modulates virus replication by a translation-independent mechanism (78). In the case of SARS-CoV-2, the RNA-binding protein RBM24 was shown to bind to the 5′-NCR to the 5′-NCR, preventing 80S ribosome assembly, which in turn inhibits polyprotein translation and virus replication (79). In another report, the Lamp2 protein was shown to bind the 5′-NCR of the SARS-CoV-2 genome. Its level was upregulated in virus-infected cells; the absence of Lamp2a enhanced the viral RNA levels, whereas its overexpression significantly reduced the viral RNA levels (80). However, the mechanism of antiviral action has not been studied. Yet another protein, pseudouridine synthase 4 (Pus4), was shown to bind specifically to the 3′-NCR of the brome mosaic virus RNA (81). Here, the protein binding interfered with the plus-sense genomic RNA synthesis, reducing virus replication. This is similar to the present report showing specific binding of EWSR1 to the 3′-NCR of the plus-sense CHIKV genomic RNA and the 3′-end of the replication intermediate RNA, reduced plus- and minus-sense genomic RNA synthesis in the presence of overexpressed EWSR1, and reduced virus replication.

Our studies have shown that both CHIKV nsP2 and EWSR1 bind the 3NCR48 sequence. CHIKV nsP2 helicase and the replicase complex must move along the genomic RNA template for its unwinding and copying. EWSR1 binding to 3NCR48 could sterically hinder or physically block CHIKV nsP2 and the viral replicase complex access and assembly, or movement, at the 3′-end of the CHIKV plus-sense genomic RNA, thus negatively modulating the minus-sense RNA synthesis. Similarly, EWSR1 binding to the 5NCR-RC50 sequence could obstruct the access and movement of CHIKV nsP2 and the viral replicase at the 3′-end of the replication-intermediate minus-sense CHIKV RNA, inhibiting the synthesis of the plus-sense CHIKV genomic RNA, resulting in the inhibition of virus replication. We have also shown that EWSR1 binds to CHIKV nsP2, resulting in the inhibition of its helicase activity. EWSR1 at the viral RNA replication site may bind CHIKV nsP2, making it unavailable for RNA replication, resulting in the inhibition of viral RNA synthesis and reduced virus titers. This presents an alternate mechanism for the EWSR1-mediated antiviral effect seen during CHIKV replication. How, and if at all, either or both mechanisms contribute to CHIKV replication inhibition remains to be studied further.

The 3′-NCR’s 48-nucleotide conserved sequence represented in the 3NCR48 RNA has been predicted to form a hairpin secondary structure (15, 82, 83). EWSR1 binding may stabilize or destabilize such key RNA elements, altering local secondary or tertiary RNA structures and thereby affecting the efficiency of replication. EWSR1 could help recruit host factors involved in RNA decay or restrict viral RNA from protective compartments, leading to enhanced viral RNA degradation. The role of this mechanism in EWSR1-mediated inhibition of CHIKV replication, if any, needs further investigation.

We have studied the role of EWSR1 in human cell lines such as HEK293T and HAP1. CHIKV replicates in mosquitoes, completing its lifecycle through midgut infection (84, 85). While orthologs of EWSR1 have been reported across diverse animal species, primarily vertebrates, fish, and birds in UniPROT (A0A7L4K950_9AVES, A0A7L3QWB7_9SYLV, A0A6J2USF1_CHACN, A0A7L4ALF1_9AVES), no orthologs have been reported in mosquitoes so far. It may be interesting to identify EWSR1 orthologs and explore their role in CHIKV replication in mosquito cell lines, which are efficiently infected by the virus (86).

## Data Availability

All data related to this work are included in the article, other than the raw mass spectrometry data, which have been deposited to the Indian Biological Data Centre (IBDC) at the Indian Proteome Databank (IPD) portal (accession ID: IPD 9067).

## SUPPLEMENTAL MATERIAL

Supplemental material jvi.00141-26-s0001.pdf Fig. S1; Tables S1 and S2.

ASM does not own the copyrights to Supplemental Material that may be linked to, or accessed through, an article. The authors have granted ASM a non-exclusive, world-wide license to publish the Supplemental Material files. Please contact the corresponding author directly for reuse.
