# Supplementary material for: Ewing sarcoma breakpoint region 1 (EWSR1) protein binds to nonstructural protein 2 and non-coding regions of chikungunya virus genome to inhibit virus replication
Source: J Virol. 2026 Jul 21;100(8):e00141-26. doi: 10.1128/jvi.00141-26 (PMC13483453; doi:10.1128/jvi.00141-26)
Supplement: Supplementary file 1 [file jvi.00141-26-s0001.pdf]

Figure S1

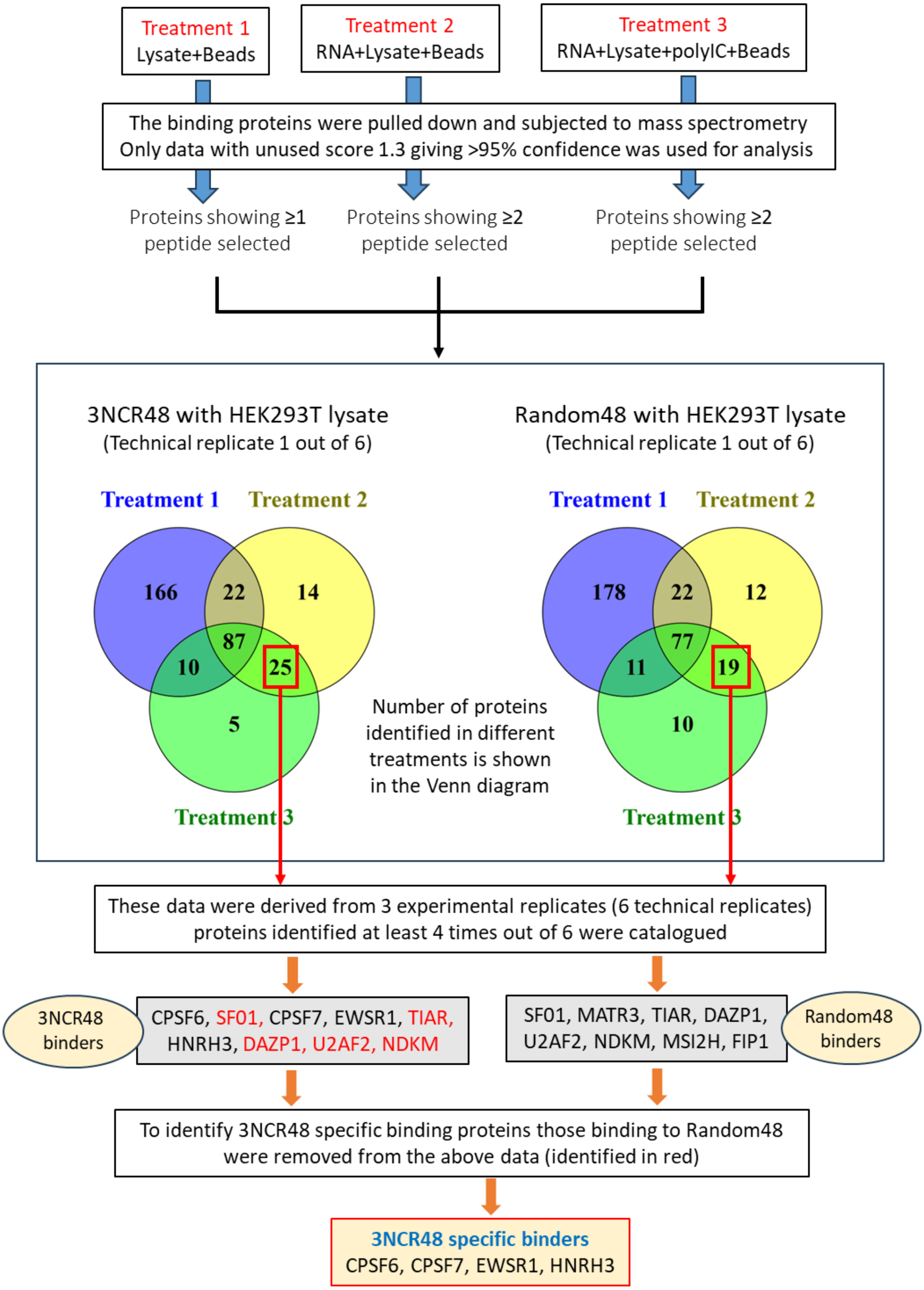

Figure S1. Identification of the host proteins binding to the CHIKV RNAs.
HEK293T, ERMS, or Vero cell lysate was incubated in binding buffer with streptavidin-labeled synthetic CHIKV RNA with or without polyIC. The RNA protein complexes were pulled down using streptavidin-labelled beads and analyzed by mass spectrometry. Using data from 3 independent experiments having 2 technical replicases each, CHIKV RNA specific binders were identified by subtracting those binding to a non-specific RNA Random48 (Table 1). The illustration above shows identification of the 3NCR48 RNA binding proteins, as an example.

Table S1

Table S1: Nucleotide sequence of the synthetic RNAs

RNA	Nucleotide sequence (5’ to 3’)
3NCR40	GUAGUUCAAAGGGCUAUAAAACCCCUGAAUAGUAACAAAA
3NCR48	UCCGAACCCACAGGGACGUAGGAGAUGUUAUUUUGUUUUUAAUAUUUC
5NCR51	GCAGGUCACACCGAAUGACCAUGCUAAUGCUAGAGCGUUCUCGCAUCUAGC
5NCR-RC50	UCUCUUGCUUUGCAGAGUAGAGCAGUAAGAAACUGGUAGGCUACGUGUGU
Random48	GUGAUUCAGAGGUUUUUGACUUUAGUAAAUCGCCCAUCCUUUGAUAGA

The G/U and A/U rich 14-mer sequence at the 3’-end of 3NCR48 is underlined.

Table S2

Table S2. Nucleotide sequence conservation.

	CHIKV1 (JF274082.1)	CHIKV2 (MH349097.1)	CHIKV3 (EU224269.1)	BFV (MK697274.1)	RRV1 (GQ433359.1)	RRV2 (NC_075016.1)	ONNV1 (M20303.1	ONNV2 (NC_001512.1)	GETV1 (LC814433.1)	GETV2 (MT086508.1)
3NCR48	100	100	100	82.6	82.6	80.4	89.2	89.1	78.7	78.7
3NCR40	100	100	100	97.5	97.5	97.4	56.8	56.8	97.5	97.5
5NCR51	100	97.6	100	75	93.8	93.8	87.2	87.5	93.8	93.8
5NCR-RC50	100	100	100	60	57.4	59.3	86	85.4	62.3	62.3

3NCR48, 3NCR40, 5NCR51, and 5NCR-RC50 RNA sequences were aligned with the genomic RNA sequences of different alphaviruses listed above (GenBank accession number in brackets) using the LALIGN software. Percent similarity of the sequence is shown in the table.
